# Efficient Prediction of Multicomponent Adsorption Isotherms and Enthalpies of Adsorption in MOFs Using Classical Density Functional Theory

**DOI:** 10.1021/acs.jpcb.5c08035

**Published:** 2026-02-05

**Authors:** Nadine Thiele, Tiong Wei Teh, Benjamin Bursik, Marcel Granderath, Gernot Bauer, Vincent Dufour-Décieux, Philipp Rehner, Rolf Stierle, André Bardow, Niels Hansen, Joachim Gross

**Affiliations:** † Institute of Thermodynamics and Thermal Process Engineering, 9149 University of Stuttgart, Pfaffenwaldring 9, 70569 Stuttgart, Germany; ‡ Energy & Process Systems Engineering, Department of Mechanical and Process Engineering, 27219 ETH Zurich, Tannenstrasse 3, 8092 Zurich, Switzerland

## Abstract

We demonstrate that classical density functional theory (DFT) based on the PC-SAFT equation of state is a fast, accurate, and predictive model to predict multicomponent adsorption in porous materials, which is an essential step toward the design of next-generation adsorbents for relevant applications. Using GPU acceleration, adsorption isotherms and adsorption enthalpies can be obtained in a matter of seconds, which is several orders of magnitude faster than grand canonical Monte Carlo (GCMC) simulations. Using metal–organic frameworks as adsorbents and non- or weakly polar molecules as adsorbates, we validate our approach by performing GCMC simulations for binary, ternary, and quaternary mixtures with practically relevant applications, such as noble gas separations (Kr/Xe, Ar/Kr/Xe), direct dry air capture (CO_2_/N_2_), hydrogen enrichment (CH_4_/H_2_, CH_4_/H_2_/N_2_) and adsorbed natural gas (CH_4_/C_3_H_8_, CH_4_/C_2_H_6_/C_3_H_8_, CH_4_/C_2_H_6_/C_3_H_8_/N_2_). Classical DFT reproduces loadings and adsorption enthalpies of the mixtures in close agreement with results from GCMC simulations. Thus, classical DFT expands our toolbox for studying multicomponent adsorption.

## Graphical abstract

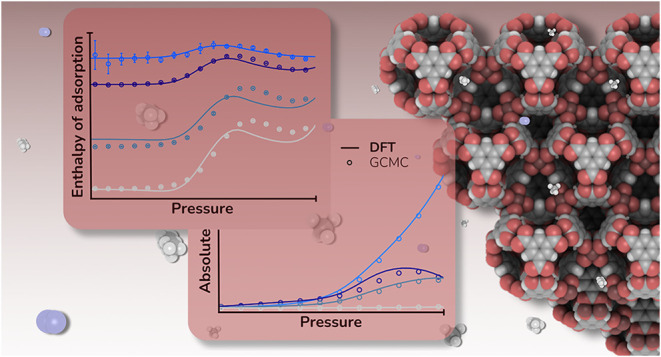

## Introduction

In recent years, adsorption processes have gained attention as promising routes for separation and storage applications.^1,2^ For many separations, adsorption processes can substantially reduce the energy demand compared to state-of-the-art processes such as cryogenic distillation.^3^ Promising porous materials have emerged, such as zeolites, metal–organic frameworks (MOFs), and covalent organic frameworks (COFs). These materials offer large surface areas and high tunability.^1^ However, this tunability corresponds to a vast material design space, making its experimental exploration slow and resource-intensive.

The most promising candidates can be identified efficiently using computational screening, and can then be synthesized and experimentally characterized.^4,5^ For this purpose, adsorption properties, i.e., adsorption isotherms and enthalpies of adsorption, need to be computed.^4,6,7^ These properties are typically determined through molecular simulations, such as grand canonical Monte Carlo (GCMC) simulations,^6,8,9^ which have been shown to successfully match experimental results if a meaningful force field is available.^4,10,11^ This predictive capability has enabled large-scale computational screening of porous materials using GCMC.^6,12,13^

Assessing the separation performance requires the evaluation of mixture properties. In material screenings, the mixture properties are either used to characterize the adsorption behavior (e.g., selectivity)^14^ or for process-level evaluation (e.g., energy demands).^4^ Evaluating mixture adsorption across a wide range of conditions (temperature, pressure, and composition) with GCMC is computationally cumbersome. Therefore, GCMC is commonly used for computing selected pure component isotherms. Temperature extrapolation can then be performed by applying the Clausius–Clapeyron equation, while the extended dual-site Langmuir^15,16^ isotherm or the ideal adsorbed solution theory (IAST) deliver mixture properties.^4,7^ For proper fitting an extrapolation model for mixtures, the pure-component isotherms require many points, preferably from the Henry regime to the saturation regime.^7^ This requirement and the high computational cost of computing pure-component isotherms with GCMC^17^ lead to only thousands of materials being screened at the process level^4^ which is small compared to the quasi-infinite design space of porous materials.

To evaluate the energy demand of a separation and to extrapolate isotherms to other temperatures based on the Clausius–Clapeyron equation in process simulations, the enthalpy of adsorption is a crucial factor.^18^ However, in a screening context, enthalpies of adsorption can display high statistical uncertainty because running long GCMC simulations to obtain equilibrated enthalpies of adsorption is computationally expensive.^17^ This uncertainty can propagate to the process simulation results, affecting the predicted process performance.

In recent years, classical density functional theory (DFT) has emerged as a promising alternative to GCMC for predicting adsorption properties.^17,19–24^ Several variants of classical DFT exist: One- (1D) and two-dimensional (2D) classical DFT simplify the adsorbent structure and require parameter fitting to either experiments or simulations, making them useful primarily for extrapolating to new conditions.^25,26^ In contrast, three-dimensional classical DFT (3D-DFT) is a fully predictive approach. It requires the same inputs as GCMC – a structure representation (e.g., a Crystallographic Information File (CIF)), a force field for the adsorbent, and a description of fluid interactions – making it a self-contained method for studying adsorption in porous materials. Extensive studies demonstrate that 3D-DFT can reproduce GCMC isotherms with high accuracy for molecules that are well-described by single-site models (e.g., methane, hydrogen), as well as for small molecules with modest Coulombic interactions (e.g., nitrogen, ethane).^17,20–22,24,27,28^ 3D-DFT also provides reasonable accuracy for larger molecules (e.g., butane)^19,20^ and those with stronger electrostatic interactions (e.g., carbon dioxide).^17^ In terms of efficiency, 3D-DFT is two to 3 orders of magnitude faster than GCMC when run on CPUs,^17^ and recent GPU implementations achieve even greater speedups.^20,29^ In this context, classical DFT always describes 3D-DFT.

Validation of classical DFT for mixture adsorption isotherms remains, however, relatively scarce in the literature. Sauer and Gross^30^ predicted argon/krypton mixtures in slit and cylindrical pores and showed good agreement with GCMC and IAST. They also investigated methane/*n*-butane mixtures in slit pores, where they pointed out limitations of classical DFT at low pressure. Liu et al.^31^ extended the validation to real adsorbents by comparing DFT and GCMC for carbon dioxide/methane and carbon dioxide/nitrogen mixtures in two MOFs, treating all molecules as single-site models, and reported good agreement between the methods. Kessler et al.^19^ investigated methane/ethane and methane/*n*-butane mixtures in COFs, observing near-perfect agreement for methane/ethane and good agreement for methane/*n*-butane. Sang et al.^32^ assessed methane/ethane mixtures in two MOFs, comparing GCMC and classical DFT, and found the level of agreement to vary with pressure, temperature, and composition. Good agreement between GCMC and classical DFT for methane/ethane mixtures was also found in cationic Faujasites.^20^ However, a comprehensive validation of classical DFT for mixtures – across multiple real porous material structures and gas compositions, while accounting for computational cost – is missing in the literature.

The present work systematically assesses classical DFT for the adsorption of binary, ternary, and even quaternary mixtures in several representative structures. We present cases where classical DFT performs well, as well as its current limitations, providing guidance on its applicability for future studies.

## Modeling Mixture Adsorption in Porous Media

The following section introduces the three approaches, classical DFT, GCMC, and IAST, used to calculate mixture adsorption in the present work.

### Classical Density Functional Theory Based on PC-SAFT

Classical DFT is a powerful tool for modeling inhomogeneous fluids, including density profiles at vapor–liquid interfaces or fluid–solid interfaces, such as those that occur in adsorption. It is based on the grand canonical ensemble, wherein the chemical potentials **μ** = {μ_*i*_,..., μ_*N*_c__} of all components *i*, the system volume *V*, and the temperature *T* remain constant. The grand canonical potential functional, Ω, depends on the inhomogeneous density profiles, **ρ** = {ρ_1_(**r**),..., ρ_*N*_c__(**r**)}, with the spatial coordinate **r**, given as

1 $$\Omega \left[\rho \left(r\right)\right]=F\left[\rho \left(r\right)\right]+\sum_{i=1}^{N_{c}}\int \rho_{i}\left(r\right)\left(V_{i}^{\mathrm{ext}}\left(r\right)-\mu_{i}\right)dr$$

where *F*[**ρ**(**r**)] is the intrinsic Helmholtz energy functional, accounting for fluid–fluid interactions, and *V*_*i*_^ext^(**r**) is the external potential, capturing fluid–solid interactions imposed by the porous media in case of adsorption. Square brackets indicate functional dependence. Bold symbols represent vectors of the respective quantities existing for all components from *i* = 1··· *N*_c_.

At thermodynamic equilibrium, the grand potential density functional is minimal for the equilibrium density profile **ρ** ^0^(**r**), and its value is equivalent to the grand canonical potential

2 $$\Omega \left[\rho \left(r\right)\neq \rho^{0}\left(r\right)\right]>\Omega \left[\rho^{0}\left(r\right)\right]=\Omega \left(\mu ,V,T\right)$$

so that the functional derivative with respect to the density profiles vanishes. This leads to the Euler–Lagrange equation, the main equation of classical DFT, given as

3 $${\frac{\delta \Omega \left[\rho \right]}{\delta \rho_{i}\left(r\right)}|}_{\rho \left(r\right)=\rho^{0}\left(r\right)}=\frac{\delta F\left[\rho \right]}{\delta \rho_{i}\left(r\right)}+V_{i}^{\mathrm{ext}}\left(r\right)-\mu_{i}=0\forall i$$

For simplicity, the superscript 0 in the equilibrium density profile will be omitted in the following, so that **ρ**(**r**) will refer to the set of equilibrium density profiles for all components in the system.

In this work, the fluid–fluid interactions are described by the intrinsic Helmholtz energy functional, *F*[**ρ**(**r**)], based on the PC-SAFT equation of state.^33,34^ In the PC-SAFT model, fluid molecules are described as chains of *m*_*i*_ tangentially bonded spherical segments. Spherical molecules correspond to *m*_*i*_ = 1, while nonspherical molecules are represented by *m*_*i*_ > 1. Each segment is characterized by a size parameter σ_*ii*_ and an energy interaction parameter ε_*ii*_. The PC-SAFT parameters used in this work were previously adjusted to vapor–liquid equilibrium data^35^ and are listed in Table S1 in the Supporting Information. This makes results for inhomogeneous systems from classical DFT purely predictive. To determine the parameters for the molecular binary interactions of fluids *i* and *j*, the Lorentz^36^ and extended Berthelot^37^ combining rules are used, written as

4 $$\sigma_{\mathrm{ij}}=\frac{\sigma_{\mathrm{ii}}+\sigma_{\mathrm{jj}}}{2}$$

5 $$\varepsilon_{\mathrm{ij}}=\sqrt{\varepsilon_{\mathrm{ii}}\varepsilon_{\mathrm{jj}}}\left(1-k_{\mathrm{ij}}\right)$$

where for some of the mixtures considered in this work, a binary interaction parameter *k*_*ij*_ is introduced. For many pairs, however, the binary interaction parameter is set to zero and the Berthelot rule is recovered. In previous work,^38^ the binary interaction parameter *k*_*ij*_ was adjusted to experimental phase equilibrium data. The binary interaction parameters for the PC-SAFT model used in this work are listed in Table S2 in the Supporting Information.

Similarly to the PC-SAFT equation of state, the Helmholtz energy functional can be divided into several additive contributions. In this work, we only consider nonpolar and nonassociating molecules. Accordingly, the Helmholtz energy functional can be expressed as

6 $$F\left[\rho \left(r\right)\right]=F^{\mathrm{ig}}\left[\rho \left(r\right)\right]+F^{\mathrm{res}}\left[\rho \left(r\right)\right]$$

with the residual Helmholtz energy contributions

7 $$F^{\mathrm{res}}\left[\rho \left(r\right)\right]=F^{\mathrm{hs}}\left[\rho \left(r\right)\right]+F^{\mathrm{hc}}\left[\rho \left(r\right)\right]+F^{\mathrm{disp}}\left[\rho \left(r\right)\right]$$

where the hard-sphere contribution (hs) models segments as impenetrable spheres described by fundamental measure theory,^39–41^ the hard-chain contribution (hc)^42,43^ accounts for the connectivity between segments in a chain, and the dispersion contribution^44^ (disp) models van der Waals attractions. An expression for the respective contributions can be found in the corresponding references, or in the Supporting Information of Stierle et al.^45^ From statistical mechanics, the structure of the ideal gas contribution (ig) is exactly known as

8 $$\beta F^{\mathrm{ig}}\left[\rho \left(r\right)\right]=\sum_{i=1}^{N_{c}}\int \rho_{i}\left(r\right)\left(\ln\left(\rho_{i}\left(r\right)\Lambda_{i}^{3}\right)-1\right)dr$$

with the inverse temperature $\beta =\frac{1}{k_{B}T}$ and the Boltzmann constant *k*_B_ and the effective de Broglie wavelength Λ_*i*_, which includes the intramolecular partition sum. All three contributions to the residual Helmholtz energy, *F* ^res^, are expressed as weighted density approximations of the form

9 $$\beta F^{\mathrm{res}}\left[\rho \left(r\right)\right]=\int \Phi^{\mathrm{res}}\left(n_{\alpha }\left(r\right)\right)dr$$

where Φ^res^ = Φ^hs^ + Φ^hc^ + Φ^disp^ is the reduced Helmholtz energy density of the residual contributions, which are only a function of the respective weighted densities **n**_α_ = {*n*_α,*i*_,..., *n*_α,*N*_c__}, calculated from convolutions of the partial density profiles with weight functions ω_*i*_^α^, according to

10 $$n_{\alpha }=\sum_{i=1}^{N_{c}}n_{\alpha ,i}\left(r\right)=\sum_{i=1}^{N_{c}}\int \rho_{i}\left(r^{\prime }\right)\omega_{i}^{\alpha }\left(r-r^{\prime }\right)dr^{\prime }$$

Here, α represents a generic index of the respective weight function (e.g., for the hard-sphere contribution, α = 0, 1, 2, 3 denotes the scalar-valued weighted densities, and α = V1, V2 denotes the vector-valued weighted densities. A similar weight function is also determined in the dispersion term). A more detailed description can be found in the work of Stierle et al.^45^

In classical DFT, the effect of a porous material on a fluid is incorporated through the external potential, *V*_*i*_^ext^ (**r**), which is based on interaction sites of all atoms of the solid material toward a fluid species *i*. The external potential is calculated by considering the interactions of all segments of a PC-SAFT molecule *i* with all solid atoms of the porous material individually, according to

11 $$V_{i}^{\mathrm{ext}}\left(r\right)=m_{i}\sum_{s=1}^{N_{s}}4\varepsilon_{\mathrm{si}}\left({\left(\frac{\sigma_{\mathrm{si}}}{\left|r_{s}-r\right|}\right)}^{12}-{\left(\frac{\sigma_{\mathrm{si}}}{\left|r_{s}-r\right|}\right)}^{6}\right)$$

where *N*_s_ is the number of solid atom interaction sites of the porous material and **r**_s_ denotes the position of each solid atom interaction site, taken from the CIF of the solid structure, as used in molecular simulations. Prefactoring *m*_*i*_ in eq 11 and considering only an individual spherical segment of a molecule of type *i* implies an assumption: all segments are assumed to be indistinguishable. The difference of a terminal segment toward a segment in the middle of a chain is not resolved in our classical DFT model. The summation in eq 11 is constrained to atoms within a predefined cutoff radius, *r*_c_. Atoms located beyond this distance are assumed to have a negligible effect on the external potential and are therefore omitted. In this work, a cutoff radius of *r*_c_ = 14 Å is employed.

The molecular binary interaction parameters σ_*si*_ and ε_*si*_ are calculated according to eqs 4 and 5. The model parameters assigned to the atomic sites of the porous material σ_*ss*_ and ε_*ss*_ are taken from force fields designed to represent the material’s properties. In this work, parameters from the Universal Force Field (UFF)^46^ and DREIDING^47^ are employed, given in Table S3 in the Supporting Information.

When modeling adsorption, the porous material is considered to be in contact with a bulk-phase reservoir. Using the PC-SAFT equation of state, the chemical potential is therefore determined by its bulk pressure *p* ^bulk^, temperature *T*, and bulk molar fractions **x** ^bulk^ = {*x*_*i*_^bulk^,..., *x*_*N*_c__^bulk^}, so that μ_*i*_ = μ_*i*_^bulk^(*p* ^bulk^, *T*, **x** ^bulk^). An adsorption isotherm can subsequently be obtained by performing a series of classical DFT calculations at varying chemical potentials, corresponding to different bulk pressures. With the residual chemical potential μ_*i*_^res,bulk^ = μ_*i*_^res^(ρ_*i*_^bulk^) = μ_*i*_(ρ_*i*_^bulk^) – μ_*i*_^ig^(ρ_*i*_^bulk^), eq 3 leads to

12 $$\rho_{i}\left(r\right)=\rho_{i}^{\mathrm{bulk}}\exp\left(\beta \mu_{i}^{\mathrm{res},\mathrm{bulk}}-\frac{\delta \beta F^{\mathrm{res}}\left[\rho \right]}{\delta \rho_{i}\left(r\right)}-\beta V_{i}^{\mathrm{ext}}\left(r\right)\right)$$

where ρ_*i*_^bulk^ is the corresponding bulk density of component *i* and the ideal gas contribution of the chemical potential is evaluated at bulk-phase conditions, μ_*i*_^ig,bulk^ = *k*_B_ *T* ln (ρ_*i*_^bulk^ Λ_*i*_^3^). The equilibrium density profile in eq 12 is determined for each state point, and the absolute amount of adsorbed molecules, **N** ^ads^ = {*N*_*i*_^ads^,..., *N*_*N*_c__^ads^}, is obtained by integrating each equilibrium density profile over the system volume, written as

13 $$N_{i}^{\mathrm{ads}}=\int \rho_{i}\left(r\right)dr$$

Consequently, the adsorbed amount is obtained as a function of the bulk conditions and the external potential of the porous material, **N** ^ads^(*p* ^bulk^, *T*, **x** ^bulk^, **V** ^ext^). The excess adsorbed amount, *N*_*i*_^exc^, can easily be obtained by

14 $$N_{i}^{\mathrm{exc}}=\int \left(\rho_{i}\left(r\right)-\rho_{i}^{\mathrm{bulk}}\right)dr=N_{i}^{\mathrm{ads}}-V^{\mathrm{BET}}\rho_{i}^{\mathrm{bulk}}$$

with the accessible pore volume, *V* ^BET^, determined using the Brunauer–Emmett–Teller (BET) theory.^48^ Classical DFT calculations and molecular simulations do not require defining excess properties or quantities like *V* ^BET^, but these quantities allow relating results to experimental data.

#### Discretization of Classical Density Functional Theory for Efficient Calculation of Functional Derivatives Using Automatic Differentiation

The porous structures provided by CIFs are mapped onto a three-dimensional grid by discretizing the unit cell into a specified number of grid points, *N*_grid_, along each spatial direction. At each grid point, fluid molecules are modeled as *m*_*i*_ Lennard-Jones interaction sites using the PC-SAFT parameters. Their interactions with all atoms of the porous structure are evaluated as the external potential in eq 11.

To facilitate numerical computation, the functional in eq 9 is expressed in discretized form as ∫Φ^res^(**n**_α_(**r**)) d**r** ≈ Δ*V* ∑_*k*_Φ^res^(**n**_α_(**r**_*k*_)), where Δ*V* denotes the cell volume at grid position *r*_*k*_. For an equidistant grid in each spatial direction, the cell volume is identical for all *k*. The functional derivative of the residual Helmholtz energy function in eq 12 is then efficiently calculated using the backward mode automatic differentiation. Stierle et al.^29^ presented a GPU-accelerated implementation of this approach for PC-SAFT functionals, which is employed in this work. The functional derivative can be replaced by a partial derivative,

15 $$\frac{\delta \beta F^{\mathrm{res}}\left[\rho \left(r\right)\right]}{\delta \rho_{i}\left(r_{l}\right)}\approx \frac{1}{\Delta V}\frac{\partial \left(\Delta V\sum_{k}\Phi^{\mathrm{res}}\left(n_{\alpha }\left(r_{k}\right)\right)\right)}{\partial \rho_{i}\left(r_{l}\right)}\approx \frac{\partial \left(\sum_{k}\Phi^{\mathrm{res}}\left(n_{\alpha }\left(r_{k}\right)\right)\right)}{\partial \rho_{i}\left(r_{l}\right)}$$

enabling parallelized computation at each grid position **r**_*l*_. The adsorbed amount in eq 13 can then be expressed as

16 $$N_{i}^{\mathrm{ads}}\approx \Delta V\sum_{k}^{N_{\mathrm{grid}}}\rho_{i}\left(r_{k}\right)$$

To obtain the equilibrium density profile, ρ_*i*_(**r**_*k*_), we use a damped Picard iteration. The bulk density is used as the initial density profile of each state. The equilibrium density profile is considered convergent if the L2-norm

17 $$\mathrm{res}=\frac{{∥\sum_{i}^{N_{c}}\sum_{k}^{N_{\mathrm{grid}}}\left(\rho_{i}^{\mathrm{bulk}}\exp\left(\beta \mu_{i}^{\mathrm{res},\mathrm{bulk}}-\frac{\delta \beta F^{\mathrm{res}}\left[\rho \left(r_{k}\right)\right]}{\delta \rho_{i}\left(r_{l}\right)}-\beta V_{i}^{\mathrm{ext}}\left(r_{k}\right)\right)-\rho_{i}\left(r_{k}\right)\right)∥}_{2}}{\sqrt{N_{\mathrm{grid}}N_{c}}}$$

of the Euler–Lagrange eq (eq 12) is less than the tolerance of 1 × 10^–11^.

#### Enthalpy of Adsorption

The enthalpy of adsorption of a component *i*, Δ^ads^ *h*_*i*_, is an important property that characterizes fluid–solid interactions. In classical DFT, it is determined from the equilibrium density profile.

The partial molar enthalpy of adsorption is defined as

18 $$\Delta^{\mathrm{ads}}h_{i}\equiv {\left(\frac{\partial H}{\partial N_{i}}\right)}_{T,p^{\mathrm{bulk}},N_{j\neq i}}-h_{i}^{\mathrm{bulk}}$$

where $h_{i}^{\mathrm{bulk}}={\left(\frac{\partial H}{\partial N_{i}}\right)}_{T,p^{\mathrm{bulk}},N_{j\neq i}}^{\mathrm{bulk}}$ denotes the partial molar enthalpy of component *i* in the bulk phase, *H* is the enthalpy of the microporous system and *N*_*i*_ is the number of molecules. Following the derivation provided in the Supporting Information by Dufour-Décieux et al.,^17^ the enthalpy of adsorption can be calculated from

19 $$0=\sum_{j}{\left(\frac{\partial N_{i}}{\partial \mu_{j}}\right)}_{T,V,\mu_{l\neq j}}\Delta^{\mathrm{ads}}h_{j}+T{\left(\frac{\partial N_{i}}{\partial T}\right)}_{p^{\mathrm{bulk}},V,x^{\mathrm{bulk}}}$$

For discretized unit cells, the partial derivatives of loadings in eq 19 can be expressed as

20 $${\left(\frac{\partial N_{i}}{\partial \mu_{j}}\right)}_{T,V,\mu_{l\neq j}}=\int {\left(\frac{\partial \rho_{i}\left(r\right)}{\partial \mu_{j}}\right)}_{T,V,\mu_{l\neq j}}dr\approx \Delta V\sum_{k}^{N_{\mathrm{grid}}}{\left(\frac{\partial \rho_{i}\left(r_{k}\right)}{\partial \mu_{j}}\right)}_{T,V,\mu_{l\neq j}}$$

21 $${\left(\frac{\partial N_{i}}{\partial T}\right)}_{p^{\mathrm{bulk}},V,x^{\mathrm{bulk}}}=\int {\left(\frac{\partial \rho_{i}\left(r\right)}{\partial T}\right)}_{p^{\mathrm{bulk}},V,x^{\mathrm{bulk}}}dr\approx \Delta V\sum_{k}^{N_{\mathrm{grid}}}{\left(\frac{\partial \rho_{i}\left(r_{k}\right)}{\partial T}\right)}_{p^{\mathrm{bulk}},V,x^{\mathrm{bulk}}}$$

where the partial derivatives of the density profiles do not have to be determined numerically; rather they are determined from systems of linear equations, as derived in eqs S25 and S32 of the Supporting Information by Dufour-Décieux et al.,^17^ requiring only derivatives of the Helmholtz energy functional.

### Molecular Simulation

GCMC simulations using RASPA 2.0.47 ^49^ were performed to calculate adsorption isotherms. Each GCMC run for pure-component isotherms consists of 1 × 10^4^ equilibration cycles, followed by 1 × 10^5^ production cycles. For adsorption isotherms of binary, ternary, and quaternary mixtures, all GCMC runs consist of 2.5 × 10^4^ equilibration cycles, followed by 2.5 × 10^5^ production cycles. A Monte Carlo cycle corresponds to max­(20, *N*) Monte Carlo steps, where *N* is the number of adsorbate molecules.

For GCMC simulations of pure components, translation, rotation, regrow, and insertion/deletion moves were performed in the ratio (1:1:1:2). For multicomponent mixtures, translation, rotation, regrow, insertion/deletion, and identity swap moves were performed in the ratio (1:1:1:2:2).

The intermolecular interactions were modeled using the Lennard-Jones potential. The energetic parameters of DREIDING^47^ force field were used for the framework atoms. Atom types that are not covered by DREIDING were described using the UFF^46^ force field (cf. Table S3). The atomic positions of the adsorbent atoms were obtained in the CIF format from the CoRE MOF 2025 DB^16^ (cf. Table 1). The unit cells of all MOFs considered in this work are visualized in Figure S2 in the Supporting Information. All adsorbent atoms were held rigid during the GCMC simulations. Adsorbate force fields that have been assessed and accepted by comparison to vapor–liquid equilibria (VLE) were selected in this work. CO_2_ and N_2_ are modeled with TraPPE,^50^ while alkanes (CH_4_, C_2_H_6_, and C_3_H_8_) are represented using the united-atom (UA) model of TraPPE.^51^ H_2_ is described by the single-site Buch model,^52^ and noble gases (Ar, Xe, Kr) are modeled using the force field of Vrabec et al.^53^

**Table 1:** Overview of MOFs Studied in This Work, Their Corresponding Reference Codes in the CoRE MOF Database,^16^ and the Presence of Open Metal Sites

common name	CoREid	open metal site
HKUST-1	2022[Cu][tbo]3[ASR]1	yes
MOF-505	2005[Cu][nbo]3[ASR]2	no
ZIF-8	2011[Zn][sod]3[ASR]2	no
MIL-47	2010[V][bpq]3[ASR]1	yes
CALF-20	2021[Zn][dmc]3[ASR]1	no

Analogous to the classical DFT calculations, the Lennard-Jones potential was truncated at *r*_c_ = 14 Å without tail correction. Coulombic interactions were computed using Ewald summation. The partial charges of adsorbent atoms in the CoRE MOF 2025 DB^16^ are obtained from PACMAN.^54^ To ensure consistency in chemical potentials between the simulation and classical DFT, fugacity coefficients for GCMC simulations are obtained from the PC-SAFT equation of state.^33,34^

We derive the equations necessary to calculate the partial molar enthalpy of adsorption using molecular simulations. The partial molar enthalpy of adsorption of component *i* is defined as the difference between the partial molar enthalpies in the adsorbed phase and in the bulk phase. Substituting the partial molar enthalpies with *h*_*i*_ = μ_*i*_ + *Ts*_*i*_ into eq 18 yields

22 $$\Delta^{\mathrm{ads}}h_{i}=T\left(s_{i}-s_{i}^{\mathrm{bulk}}\right)$$

where the partial molar entropies in both phases can be expressed in quantities that can be sampled from molecular simulations via the total differential of the internal energy, yielding
23

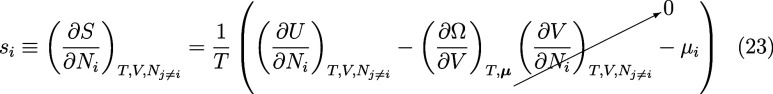

24 $$s_{i}^{\mathrm{bulk}}\equiv {\left(\frac{\partial S}{\partial N_{i}}\right)}_{T,p^{\mathrm{bulk}},N_{j\neq i}}^{\mathrm{bulk}}=\frac{1}{T}\left({\left(\frac{\partial U}{\partial N_{i}}\right)}_{T,p^{\mathrm{bulk}},N_{j\neq i}}^{\mathrm{bulk}}-p^{\mathrm{bulk}}{\left(\frac{\partial V}{\partial N_{i}}\right)}_{T,p^{\mathrm{bulk}},N_{j\neq i}}^{\mathrm{bulk}}-\mu_{i}^{\mathrm{bulk}}\right)$$

where (∂Ω/∂*V*)_*T*,**μ**_ is the grand potential density and simplifies to −*p* ^bulk^ for the bulk phase. Hereby, the partial molar enthalpy of adsorption is defined to be related to the differential entropy change in a reversible process where fluid is transferred from the bulk phase at a fixed temperature and bulk pressure to the porous material at fixed volume and temperature.^55^

Substituting eqs 23 and eq 24 into eq 22 yields

25 $$\Delta^{\mathrm{ads}}h_{i}=\underset{\mathrm{from}\;\mathrm{GCMC}}{\underset{︸}{{\left(\frac{\partial U}{\partial N_{i}\;}\right)}_{T,V,N_{j\neq i}\;}\;}}-\left(\underset{\mathrm{from}\;\mathrm{NPT}\;\mathrm{simulation}}{\underset{︸}{{\left(\frac{\partial U}{\partial N_{i}\;}\right)}_{T,p^{\mathrm{bulk}}\;,N_{j\neq i}\;}^{\mathrm{bulk}}\;+p^{\mathrm{bulk}}\;{\left(\frac{\partial V}{\partial N_{i}\;}\right)}_{T,p^{\mathrm{bulk}}\;,N_{j\neq i}\;}^{\mathrm{bulk}}\;}}\right)$$

where the partial derivatives of the adsorbed phase and the bulk phase can be calculated using GCMC and NPT simulations, respectively. The partial derivative of the adsorbed phase can be calculated from ensemble fluctuations of total energies and of the number of molecules in a single GCMC simulation,^56,57^

26 $${\left(\frac{\partial \left\langle U\right\rangle }{\partial \left\langle N_{i}\right\rangle }\right)}_{T,V,N_{j\neq i}}=\sum_{k}^{N_{c}}\frac{{\left(\frac{\partial \left\langle U\right\rangle }{\partial \mu_{k}}\right)}_{T,V,\mu_{j\neq k}}}{{\left(\frac{\partial \left\langle N_{i}\right\rangle }{\partial \mu_{k}}\right)}_{T,V,N_{j\neq i}}}=\sum_{k}^{N_{c}}\frac{\langle U\cdot N_{k}\rangle_{\mu }-\langle U\rangle_{\mu }\langle N_{k}\rangle_{\mu }}{\langle N_{i}N_{k}\rangle_{\mu }-\langle N_{i}\rangle_{\mu }\langle N_{k}\rangle_{\mu }}$$

where the angular brackets ⟨··· ⟩_**μ**_ denote the ensemble average of a GCMC simulation.

For the simplest case of a pure component adsorption (dropping the component index *i*), eq 26 becomes

27 $${\left(\frac{\partial U}{\partial N}\right)}_{T,V}=\frac{\langle U\cdot N\rangle_{\mu }-\langle U\rangle_{\mu }\langle N\rangle_{\mu }}{\langle N^{2}\rangle_{\mu }-\langle N\rangle_{\mu }^{2}}$$

For the bulk phase of the pure component, the molar enthalpy from an NPT simulation is

28 $$h^{\mathrm{bulk}}=\frac{\langle U\rangle_{\mathrm{NPT}}}{N^{\mathrm{bulk}}}+p^{\mathrm{bulk}}\frac{\langle V\rangle_{\mathrm{NPT}}}{N^{\mathrm{bulk}}}$$

where the angular brackets ⟨··· ⟩_NPT_ denote the ensemble average of an NPT simulation.

For multicomponent mixtures in the bulk phase, the calculation of partial molar enthalpies requires either the evaluation of fluctuation formulas or the numerical evaluation of the partial derivatives by a central difference scheme

29 $${\left(\frac{\partial U}{\partial N_{i}}\right)}_{T,p^{\mathrm{bulk}},N_{j\neq i}}^{\mathrm{bulk}}=\frac{{\left\langle U\left(N_{i}^{\mathrm{bulk}}+1,N_{j}^{\mathrm{bulk}}\right)\right\rangle }_{\mathrm{NPT}}-{\left\langle U\left(N_{i}^{\mathrm{bulk}}-1,N_{j}^{\mathrm{bulk}}\right)\right\rangle }_{\mathrm{NPT}}}{2}$$

A mixture of *N*_c_ components would require at least 2*N*_c_ number of NPT simulations for each thermodynamic state. To avoid the large computational cost of calculating partial molar quantities using molecular simulations, we use the PC-SAFT equation of state.^33,34^ We validated for pure components that GCMC + PC-SAFT predicts the enthalpy of adsorption with good agreement compared to results from molecular simulations (see Figures S9 and S10 in the Supporting Information).

The total system energy in the adsorbed phase, *U*, consists of the fluid–solid, fluid–fluid, and solid–solid interactions. This implies that the enthalpy of adsorption is the sum of the various contributions: First, the fluid–solid interactions that exist in the confinement but not in bulk; second, the contribution accounting for different conformational energies of the molecules in the adsorbed phase compared to the bulk phase, and last, the contribution due to framework deformation caused by mechanical stress induced during adsorption. The last term is zero under the assumption of rigid frameworks. Calculating the partial molar enthalpy of adsorption using both GCMC and NPT simulations as stated in eq 25 considers the difference in conformational energies of the molecules possessing internal degrees of freedom (DoF). However, since the PC-SAFT equation of state accounts for internal DoF only in the effective de Broglie wavelength in the ideal gas term, the intramolecular energy of the molecules must be explicitly calculated. We get

30 $$\Delta^{\mathrm{ads}}h_{i}=\underset{\mathrm{GCMC}}{\underset{︸}{{\left(\frac{\partial U}{\partial N_{i}\;}\right)}_{T,V,N_{j\neq i}\;}\;}}-\left(\underset{\mathrm{PC}-\mathrm{SAFT}}{\underset{︸}{h_{i}^{\mathrm{res},\mathrm{bulk}}\;}}+\underset{\mathrm{NVTsimulation}}{\underset{︸}{\frac{\left\langle U^{\mathrm{intra}}\;\right\rangle }{N_{i}^{\mathrm{ig}}}}}+\mathrm{RT}\right)$$

The enthalpy of the bulk phase calculated from the PC-SAFT equation of state is here defined as a residual quantity, *h*_*i*_^res,bulk^ = *h*_*i*_^bulk^ – *h*_*i*_^ig,bulk^. Because the energy *U*, however, contains energy contributions from intramolecular degrees of freedom, we subtract an average of the intramolecular energies ⟨*U* ^intra^⟩. A dedicated NVT simulation with *N*_*i*_^ig^ = 1 molecule in the ideal gas phase is performed to determine ⟨*U* ^intra^⟩. In our work, propane is the only molecule with intramolecular energy contributions. We assume that the intramolecular energy of propane in the bulk phase, $\frac{\left\langle U^{\mathrm{intra}}\right\rangle }{N_{i}^{\mathrm{ig}}}$ , does not vary with density and keeps the value of the ideal gas phase. Note that this assumption may not hold for molecules with several internal DoF.

The intramolecular energy ⟨*U* ^intra^⟩ for propane is calculated using an NVT simulation of one propane molecule in a box of 30 Å × 30 Å × 30 Å. The NVT simulation is sampled for 5000 equilibration and 50 000 production cycles with translation, rotation, and regrow moves in the ratio (1:1:1).

Since RASPA, using standard settings, assumes the bulk phase as an ideal gas phase (*H* = *U* + *pV* = *U* + *RT*) when calculating the partial molar enthalpy of adsorption using GCMC simulations, the final equation of calculating partial molar enthalpies of adsorption in this work while considering nonideal gas bulk phase is

31 $$\Delta^{\mathrm{ads}}h_{i}=\underset{\mathrm{GCMC}\;\mathrm{in}\;\mathrm{RASPA}2}{\underset{︸}{{\left(\frac{\partial U}{\partial N_{i}\;}\right)}_{T,V,\mu }\;-\mathrm{RT}\;}}-\left(\underset{\mathrm{PC}-\mathrm{SAFT}}{\underset{︸}{h_{i}^{\mathrm{res},\mathrm{bulk}}\;}}+\underset{\mathrm{NVT};\;=\;0\;\mathrm{if}\;\mathrm{no}\;\mathrm{internal}\;\mathrm{DoF}}{\underset{︸}{\frac{\left\langle U^{\mathrm{intra}}\;\right\rangle }{N_{i}^{\mathrm{ig}}}}}\right)$$

### Ideal Adsorbed Solution Theory

IAST is a model for calculating mixture adsorption properties.^58^ IAST calculations employ pure-component isotherms for each component that are fitted with an analytic isotherm model. Key assumptions of IAST are

1. The adsorbed phase can be described as an ideal solution of the adsorbed components.
2. All adsorption sites are equally accessible to all guest molecules.
3. The surface area of the guest molecules does not change upon mixing the molecules.

While other mixture adsorption models, such as the extended dual-site Langmuir, are often used in the literature,^15,16^ Moubarak et al.^7^ show that IAST leads to more robust fitting and better agreement with GCMC simulations. They also show that for reliable IAST calculations, it is important to capture the full isotherm from the Henry regime to saturation when fitting pure component adsorption isotherms.^7^ Therefore, when generating the pure-component isotherms using GCMC simulations, we ensure that the first three points of the isotherm are in the Henry regime, i.e., depend linearly on pressure. At high pressures, we evaluate the gradient of the isotherm. If the loading does not change significantly while increasing the pressure, we assume that saturation is reached. To capture the correct fluid behavior at high pressures, we perform the IAST calculations with fugacities and not with pressures. We use pyGAPS for both, the isotherm fitting and the IAST calculations, which extends the earlier developed pyIAST package.^59,60^ The isotherms are fitted to the Langmuir, dual-site Langmuir, and triple-site Langmuir models. The model with the lowest fitting error is chosen for the IAST calculations.

## Predicting Mixture Adsorption

We investigate the adsorption of several binary, ternary, and quaternary mixtures in common porous materials in equilibrium to a gas-phase mixture of composition **x** ^bulk^. An overview is provided in Table 2. To keep the discussion concise, only selected systems are presented in the following subsections. The complete set of results is available in the Supporting Information. We use GCMC simulations to benchmark comparisons with classical DFT and IAST. All adsorption isotherms were generated at *T* = 298 K.

**Table 2:** Overview of All Investigated Mixtures, Including Their Compositions in the Bulk Phase, *x*_*i*_^bulk^, and the Considered Frameworks

components				compositions				
1	2	3	4	*x*_1_^bulk^	*x*_2_^bulk^	*x*_3_^bulk^	*x*_4_^bulk^	frameworks
Kr	Xe			0.2	0.8			HKUST-1,
				0.8	0.2			MOF-505
CH_4_	H_2_			0.1	0.9			HKUST-1,
				0.5	0.5			ZIF-8
				0.9	0.1			
CH_4_	C_3_H_8_			0.5	0.5			HKUST-1
				0.9	0.1			
CO_2_	N_2_			0.5	0.5			HKUST-1,
								MIL-47,
								CALF-20
CH_4_	H_2_	N_2_		0.33	0.33	0.34		HKUST-1,
								ZIF-8
Ar	Kr	Xe		0.16	0.65	0.19		HKUST-1
				0.31	0.25	0.44		
CH_4_	C_2_H_6_	C_3_H_8_		0.939	0.032	0.029		HKUST-1
CH_4_	C_2_H_6_	C_3_H_8_	N_2_	0.935	0.032	0.007	0.026	HKUST-1

### Binary Mixture Adsorption

Figure 1 shows the adsorption isotherms of a methane/hydrogen mixture in ZIF-8, obtained from GCMC simulations, classical DFT calculations, and from IAST with pure component GCMC simulations as input (IAST + GCMC). A comparison of classical DFT and GCMC for all pure component adsorption isotherms is given in Section 1 of the Supporting Information.

**Figure 1:**
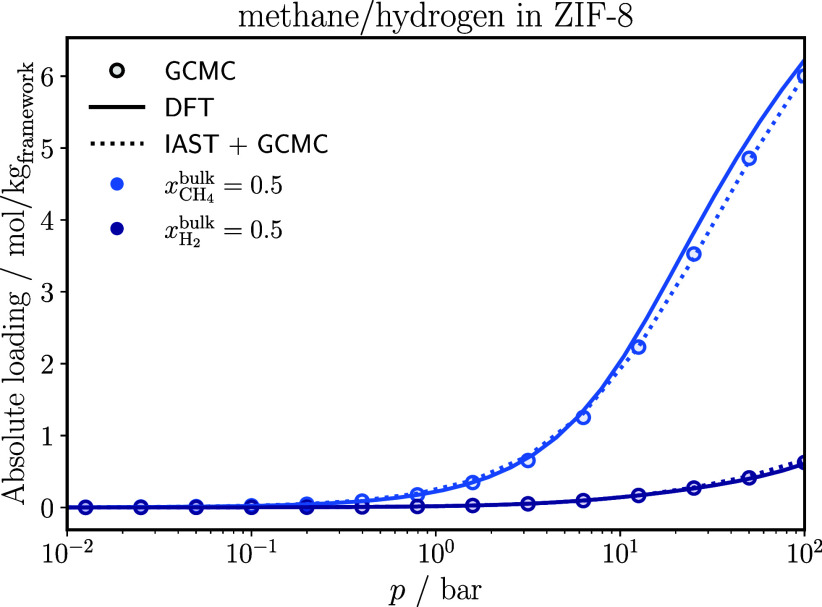
Adsorption isotherms of each component of a methane/hydrogen mixture in ZIF-8 at 298 K. The IAST results are based on Langmuir-fits on the pure component GCMC isotherms. The statistical uncertainties in the GCMC results are smaller than the symbol size.

Classical DFT shows very good agreement with GCMC simulations over the entire pressure range. This agreement is expected for this system because methane and hydrogen are both modeled as spherical molecules that can be represented as single interaction sites. In GCMC simulations, methane is modeled with the united-atom TraPPE force field,^50^ in which only the carbon center has an assigned interaction site, while hydrogen is described by the single-site Buch model.^52^ In classical DFT, based on the PC-SAFT equation of state, both components are described with a segment number of *m*_CH4_ = 1 = *m*_H2_, allowing for an unambiguous comparison to GCMC.

IAST aligns well with the GCMC results, suggesting that the adsorbed phase is approximated well by an ideal solution. However, it is important to note that IAST requires pure component adsorption isotherms from GCMC simulations as input, whereas classical DFT is purely predictive.

Although PC-SAFT parameters for hydrogen have been reported by Eller et al.,^61^ they are not suitable for the present study: These parameters were adjusted to supercritical *p*, *V*, *T*-data and are mainly suitable for the gas phase. They do not accurately predict vapor–liquid behavior. In order to achieve a good balance between the supercritical phase and the vapor–liquid equilibrium for hydrogen, we use the same parameters as in the GCMC simulations. The modified Lennard-Jones parameters by Buch^52^ are well-established. Substituting PC-SAFT parameters with Lennard-Jones parameters does not pose significant issues for spherical molecules, as demonstrated by Sauer and Gross.^30^ We note, however, that PC-SAFT is only valid for modeling hydrogen at relatively high temperatures, such as 298 K. At lower temperatures, quantum effects, which are particularly relevant for hydrogen, would need to be considered. This requires different models than PC-SAFT (e.g., the SAFT-VRQ Mie equation of state^24,62^ ). However, we neglect these effects under the conditions investigated in this work and for a consistent comparison to GCMC simulations. The excellent quality of the results justifies this approach.

Since classical DFT calculates equilibrium density profiles, the density distributions of each component in the considered porous materials can be represented similarly to those in GCMC simulations. Figure 2 compares the normalized density profiles obtained from classical DFT and GCMC simulations.

**Figure 2:**
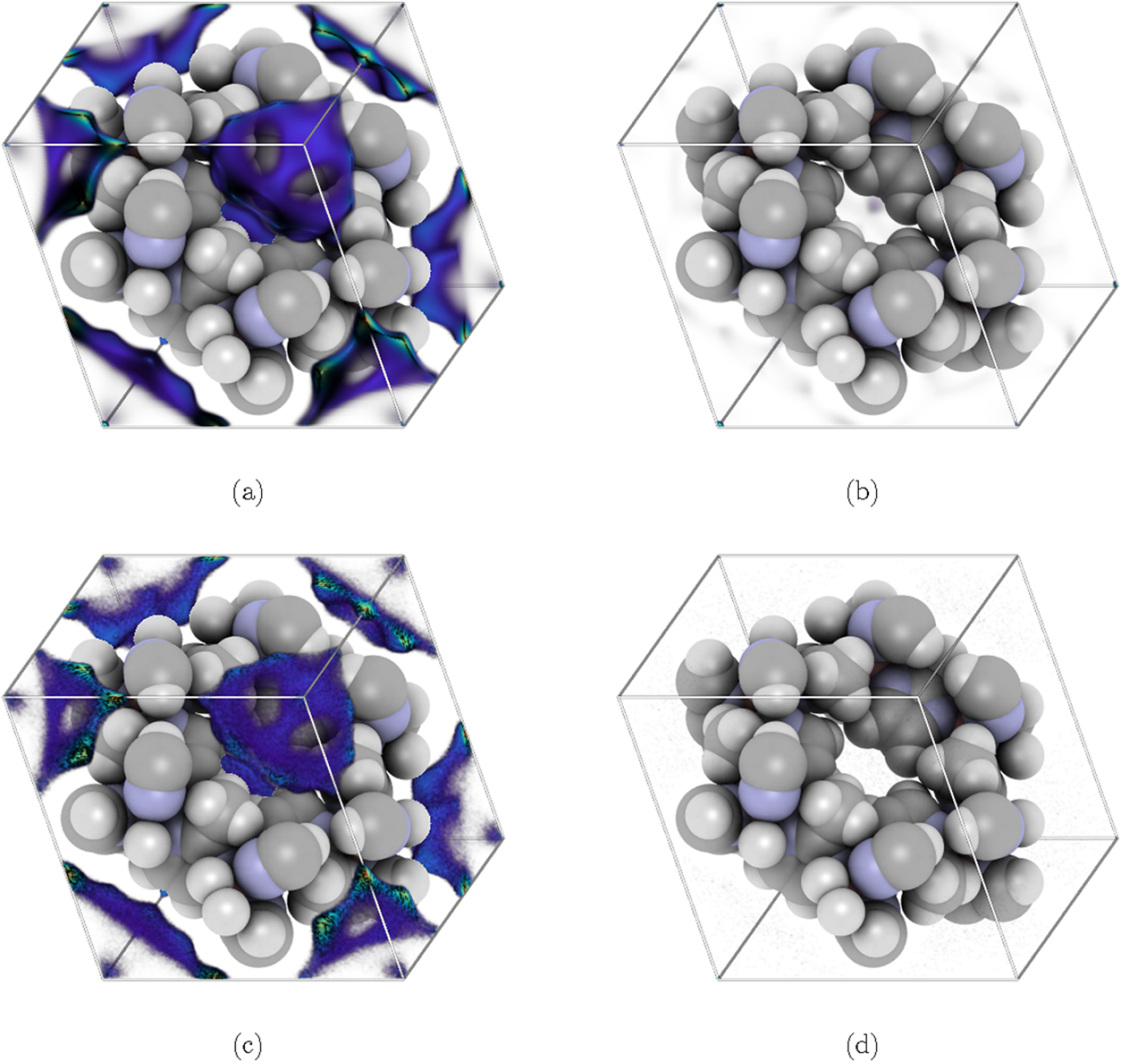
Three-dimensional normalized density profiles of each component of an equimolar methane/hydrogen mixture in ZIF-8 at 298 K and 100 bar. The results from classical DFT are shown for methane in (a) and for hydrogen in (b). For comparison, the normalized density profiles obtained from GCMC simulations, using RASPA3,^63^ are shown in (c) for methane and in (d) for hydrogen. All images are visualized with iRASPA.^64^

Figure 3 shows the adsorption isotherms of a methane/propane mixture in HKUST-1, obtained from GCMC simulations, classical DFT calculations, and from IAST + GCMC. Classical DFT accurately captures the entire pressure range for methane. For propane, classical DFT slightly overestimates the absolute loading compared to GCMC simulations in the pressure range from 2.5 × 10^–2^ bar to 3 × 10^–1^ bar, whereas it slightly underestimates the absolute loading above 3 × 10^–1^ bar. In general, we expect loss in accuracy for longer or more complex chain molecules. The deviations between classical DFT and GCMC simulations for these molecules can be traced back to how chain molecules are modeled in classical DFT, based on the PC-SAFT equation of state. Here, the connectivity of the different segments of a chain is not captured. Consequently, the local densities of segments are averaged across all segments of the chain. This simplification limits the accuracy of modeling longer or more complex chain molecules. One way to improve the model would be to implement a functional that explicitly accounts for segment connectivity and calculates the densities of individual segments (e.g., modified interfacial statistical associating fluid theory for heterosegmented chains^65–68^ ). Overall, we consider the results of classical DFT to be in good agreement to the results from GCMC simulations.

**Figure 3:**
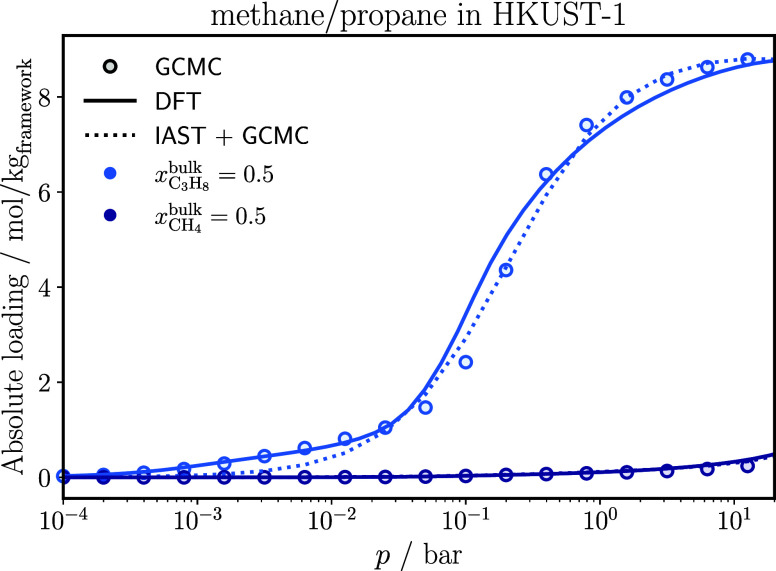
Adsorption isotherms of each component of a methane/propane mixture in HKUST-1 at 298 K. The IAST results are based on Langmuir-fits on the pure component GCMC isotherms. The statistical uncertainties in the GCMC results are smaller than the symbol size.

IAST aligns well with the GCMC simulations. However, at pressures below 2.5 × 10^–2^ bar, IAST systematically underestimates the adsorption of propane.

Figure 4 shows the adsorption isotherms of a carbon dioxide/nitrogen mixture in CALF-20, obtained from GCMC simulations, classical DFT calculations, and from IAST + GCMC. As expected, the results show that CO_2_ is preferentially adsorbed over N_2_. This is because the quadrupole moment of CO_2_ is greater than that of N_2_,^69^ which results in stronger ion-quadrupole interactions with CALF-20. Considering that, in this work, classical DFT does not explicitly account for polar interactions between the fluid molecules, as well as between the fluid and the solid, the agreement between GCMC and classical DFT is satisfactory. The objective of this work is to predict adsorption phenomena using a simple description of the Helmholtz energy functional. Accordingly, we only consider the contributions specified in eq 7 for all investigated systems. To improve the description of CO_2_ adsorption, Coulombic interactions can be included in the external potential as demonstrated in Dufour-Décieux et al.^17^ Furthermore, multipolar attraction in the fluid–fluid interactions can be included in the Helmholtz energy functional.^44^ Predictions for N_2_ remain close to the simulation data. However, classical DFT slightly underestimates the uptake in the Henry regime for both components. We note that the discrepancies between classical DFT and GCMC simulations are already apparent for both corresponding pure-component adsorption isotherms (see Figure S4 in Supporting Information). Consequently, a more accurate description of electrostatic interactions between (quadru-)­polar molecules and the porous materials is crucial to obtain more quantitative predictions in the future.

**Figure 4:**
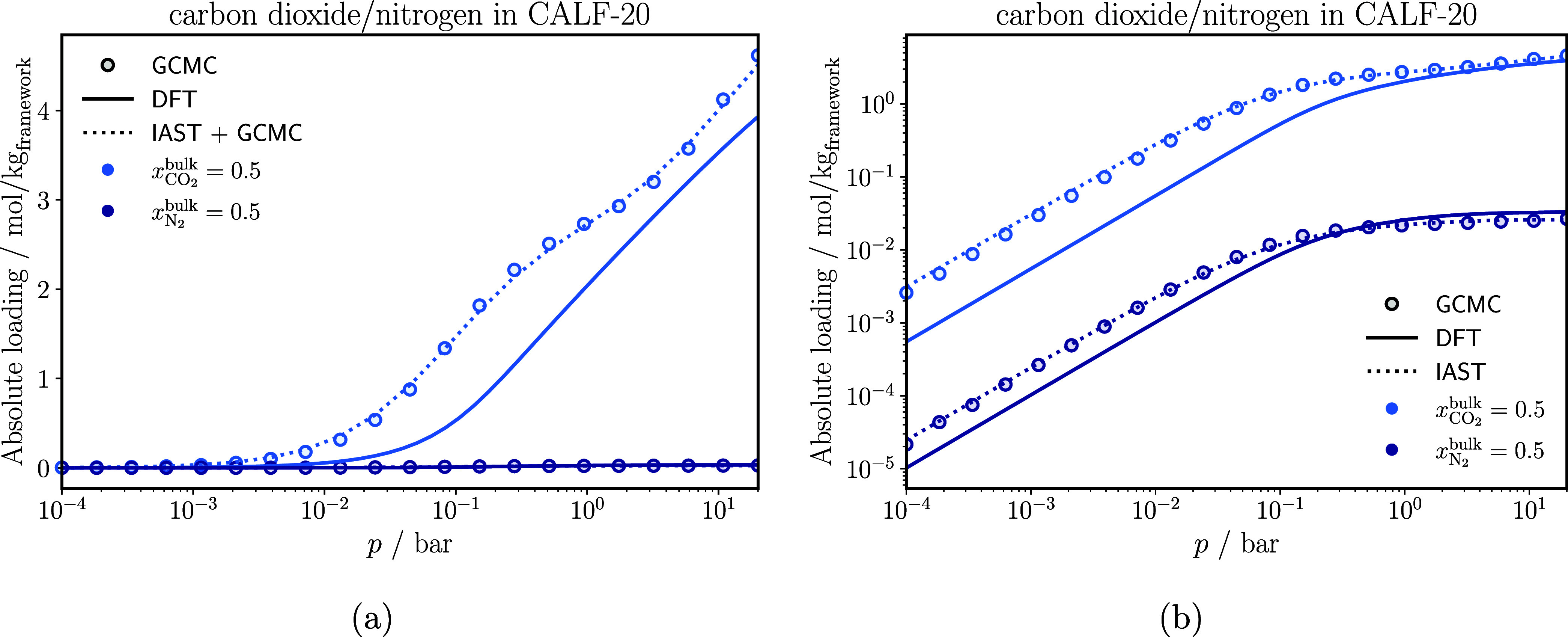
Adsorption isotherms of each component of a carbon dioxide/nitrogen mixture in CALF-20 at 298 K. The IAST results are based on Langmuir-fits on the pure component GCMC isotherms. The adsorption isotherms are displayed in a semilogarithmic diagram (a) to highlight the high-pressure behavior, and in a double-logarithmic diagram (b) to highlight the Henry regime. The statistical uncertainties in the GCMC results are smaller than the symbol size.

IAST performs well for this system and can reproduce the adsorption isotherm for both components excellently across the entire pressure range.

### Ternary and Quaternary Mixture Adsorption

While binary mixtures provide valuable insights into model performance and the fundamental adsorption mechanisms, practical separation applications typically involve gas streams with more than two components. Consequently, the analysis of mixtures with more than two components provides a more realistic evaluation of the predictive capabilities of different approaches. Compared to binary systems, ternary or quaternary mixtures introduce additional complexity due to the simultaneous competition of multiple species for adsorption sites, as well as the broader range of molecular interactions that need to be captured accurately. In the following, we discuss two representative cases: adsorption of a ternary argon/krypton/xenon mixture (see Figure 5) and adsorption of a quaternary methane/ethane/propane/nitrogen mixture (see Figure 6), both at 298 K in HKUST-1. Further results of the remaining multicomponent systems are presented in Section 2 of the Supporting Information.

**Figure 5:**
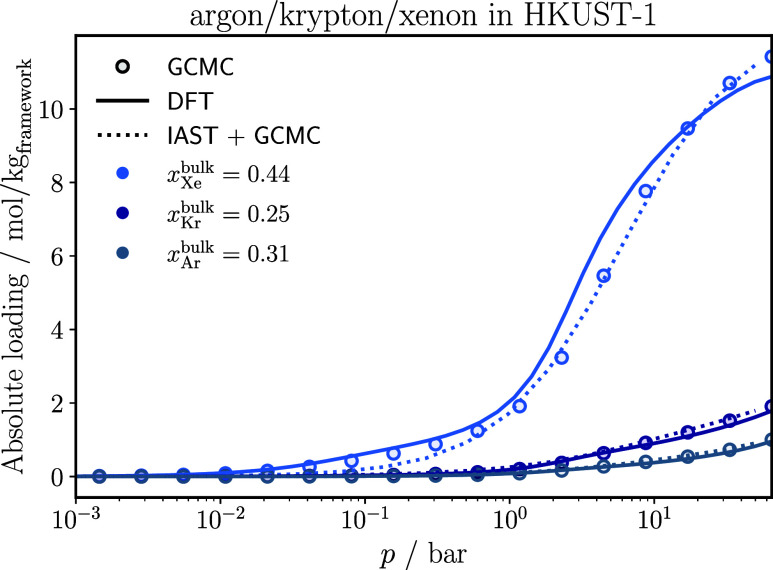
Adsorption isotherms of each component of an argon/krypton/xenon mixture in HKUST-1 at 298 K. The IAST results are based on Langmuir-fits on the pure component GCMC isotherms. The statistical uncertainties in the GCMC results are smaller than the symbol size.

**Figure 6:**
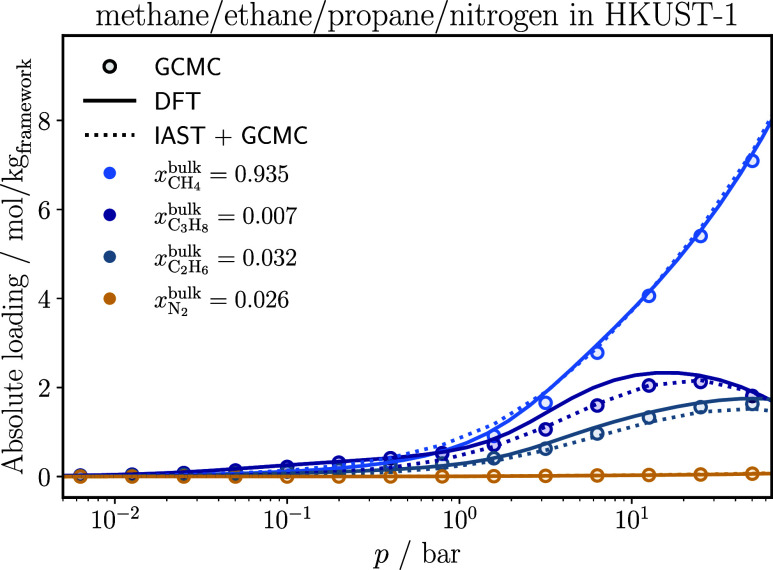
Adsorption isotherms of each component of a methane/ethane/propane/nitrogen mixture in HKUST-1 at 298 K. The IAST results are based on Langmuir-fits on the pure component GCMC isotherms. The statistical uncertainties in the GCMC results are smaller than the symbol size.

Classical DFT reproduces the GCMC results well for argon and krypton of the ternary mixture shown in Figure 5. Xenon is slightly overestimated at pressures between 1 bar and 1.5 × 10^1^ bar. Figure S1 in the Supporting Information compares the bulk VLE data for xenon from NIST^70^ with PC-SAFT and with simulations, using the force field provided by Vrabec et al.^53^ PC-SAFT accurately reproduces the VLE data from NIST.^70^ In the simulations by Vrabec et al.,^53^ deviations primarily appear in the gas phase. Thus, the difference in the adsorption isotherm between classical DFT and GCMC may already stem from the slightly different descriptions of the VLE of xenon.

For the quaternary mixture shown in Figure 6, the agreement is good for all components. Classical DFT aligns with the GCMC data across the entire pressure range and correctly predicts propane saturation.

IAST performs well for both systems shown in Figures 5 and 6, providing a reasonable description of adsorption selectivity in both mixtures.

While the results of both IAST and classical DFT align well with GCMC simulation data, the advantage of classical DFT is its computational efficiency and its solution without statistical uncertainty. Figure 7 shows the computation times for the adsorption of the pure component methane and for the previously presented ternary and quaternary systems in HKUST-1 at 298 K and at the highest and lowest pressures considered. The differences in computation time are substantial. For example, for the quaternary mixture, the GCMC simulations took approximately 5500 s at the lowest pressure and over 550000 s (more than 6 days) at the highest pressure. In contrast, the corresponding classical DFT calculations finished in about 4.46 and 4.95 s, respectively. This corresponds to a reduction in computational cost by three to 5 orders of magnitude. These results demonstrate that adsorption isotherms for both pure components and mixtures can be generated in seconds using classical DFT.

**Figure 7:**
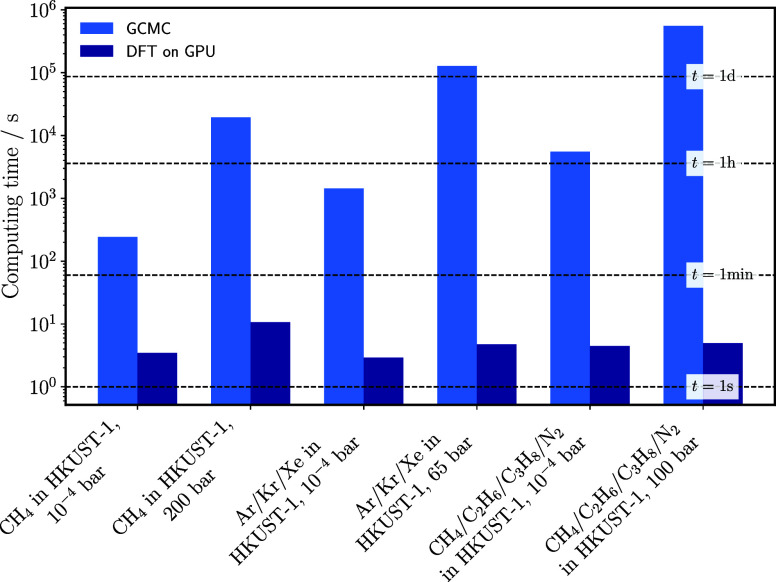
Computational cost for the calculation of adsorption at 298 K with classical DFT on a GPU compared to GCMC in RASPA 2.0.47.^49^ The computational cost of GCMC corresponds to the number of equilibration and production cycles as stated in the simulation details. Classical DFT run-times refer to calculations performed on a 64^3^ grid with a convergence tolerance of 1 × 10^–11^, using damped Picard iterations. For classical DFT, computational experiments were run on a Ubuntu 24.04.1 LTS computer, using a 3.70 GHz Intel­(R) Core­(TM) i7–8700K CPU, 32 GB RAM, and a NVIDIA GeForce RTX 4090 GPU with 24 GB VRAM. For GCMC simulations in RASPA 2.0.47, computational experiments were run on a Red Hat Enterprise Linux (RHEL) 8.4 parallel computer with distributed memory, using 2.1-GHz Intel Xeon Gold 6230 CPUs.

Although IAST itself is computationally inexpensive, it relies on pure component adsorption isotherms that are typically obtained from GCMC simulations. When this precondition is considered, IAST + GCMC calculations are more computationally expensive than classical DFT calculations.

The computational cost of a GCMC simulation depends on the number of molecules in the simulation cell and the number of Monte Carlo steps performed. This dependence is evident when comparing the lowest and highest pressures for each system. In GCMC, low pressures and high temperatures generally reduce computation time because fewer molecules are present in the system. A similar trend is observed in classical DFT because fewer iterations are needed for density profiles to converge under these conditions. The number of grid points is the most influential factor in the efficiency of classical DFT calculations. Stierle et al.^29^ demonstrated that, for the GPU implementation, computation time scales linearly with the number of grid points. Therefore, doubling the grid resolution approximately doubles the computation time. This linear scaling indicates efficient parallelization and good computational performance.

Classical DFT is particularly well-suited for applications requiring rapid and accurate adsorption predictions due to its scalability and speed compared to GCMC simulations. These applications include, e.g., high-throughput screening, or generating training data for machine learning models. The good predictive accuracy demonstrated in previous sections makes classical DFT an attractive alternative.

### Enthalpy of Adsorption

Determining adsorption enthalpies from GCMC simulations usually requires correcting for the ideal gas assumption. This correction is obtained through additional NPT simulations that calculate the partial molar bulk enthalpies of a mixture, see eq 25. While this procedure is straightforward for pure components, it becomes increasingly challenging and computationally demanding for mixtures.

To overcome this limitation, we use an alternative approach in which we obtain the partial molar enthalpies directly from the PC-SAFT equation of state with the Python package FeO_s_.^71^ This allows us to compute the required bulk reference values without the need for additional molecular simulations, thereby greatly reducing computational cost. We validated this method for pure components by comparing it to values obtained from GCMC and NPT simulations (see Figures S9 and S10 in the Supporting Information), and found good agreement. Based on this validation, we applied the same procedure to mixtures.

RASPA^49^ computes the partial molar enthalpy of adsorption based on the assumption of an ideal gas bulk phase. We correct this by replacing the ideal-gas bulk enthalpy with the PC-SAFT bulk enthalpy, as stated in eq 31. Figure 8 shows the effect of this correction for the methane/hydrogen adsorption in ZIF-8 by comparing the enthalpies of adsorption for each component obtained directly from RASPA with the corrected values.

**Figure 8:**
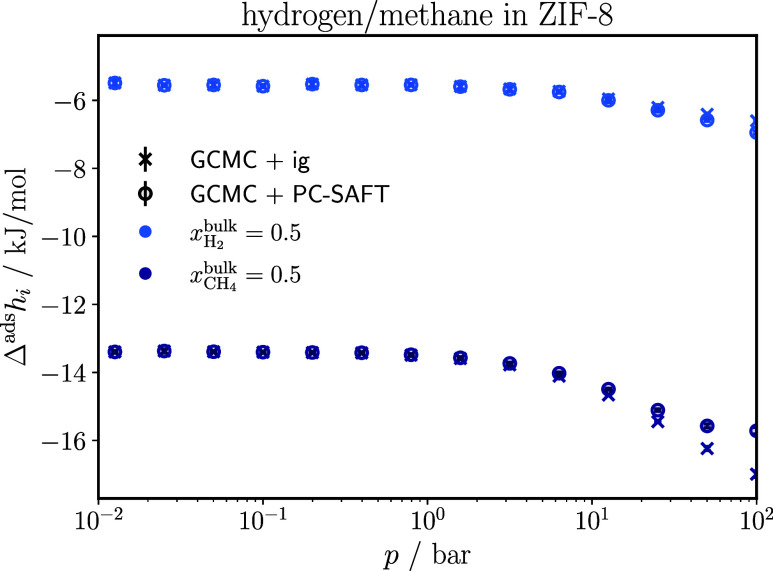
Enthalpy of adsorption for each component of a methane/hydrogen mixture in ZIF-8 at 298 K obtained directly from RASPA^49^ based on the assumption of an ideal gas bulk phase (GCMC + ig) and obtained from RASPA with the correction of a nonideal gas bulk phase (GCMC + PC-SAFT), according to eq 31.

Figure 9 shows the comparison of the enthalpy of adsorption of each component in the binary mixtures methane/hydrogen in ZIF-8, methane/propane in HKUST-1, and carbon dioxide/nitrogen in CALF-20, calculated with classical DFT and obtained from GCMC simulations and PC-SAFT (GCMC + PC-SAFT). The enthalpies of adsorption calculated with classical DFT show excellent agreement with GCMC + PC-SAFT for the methane/hydrogen mixture. For methane/propane, the agreement between GCMC + PC-SAFT and classical DFT is good. Classical DFT can reproduce the enthalpy of adsorption for both components. However, for propane, classical DFT slightly underestimates the enthalpy of adsorption in the pressure range from 2.5 × 10^–2^ bar to 3 × 10^–1^ bar. In the case of carbon dioxide/nitrogen, the agreement is satisfactory. Similar to the adsorption isotherms in Figure 4, the classical DFT calculations follow the trend of the GCMC + PC-SAFT results. However, classical DFT overestimates the enthalpy of adsorption for both CO_2_ and N_2_.

**Figure 9:**
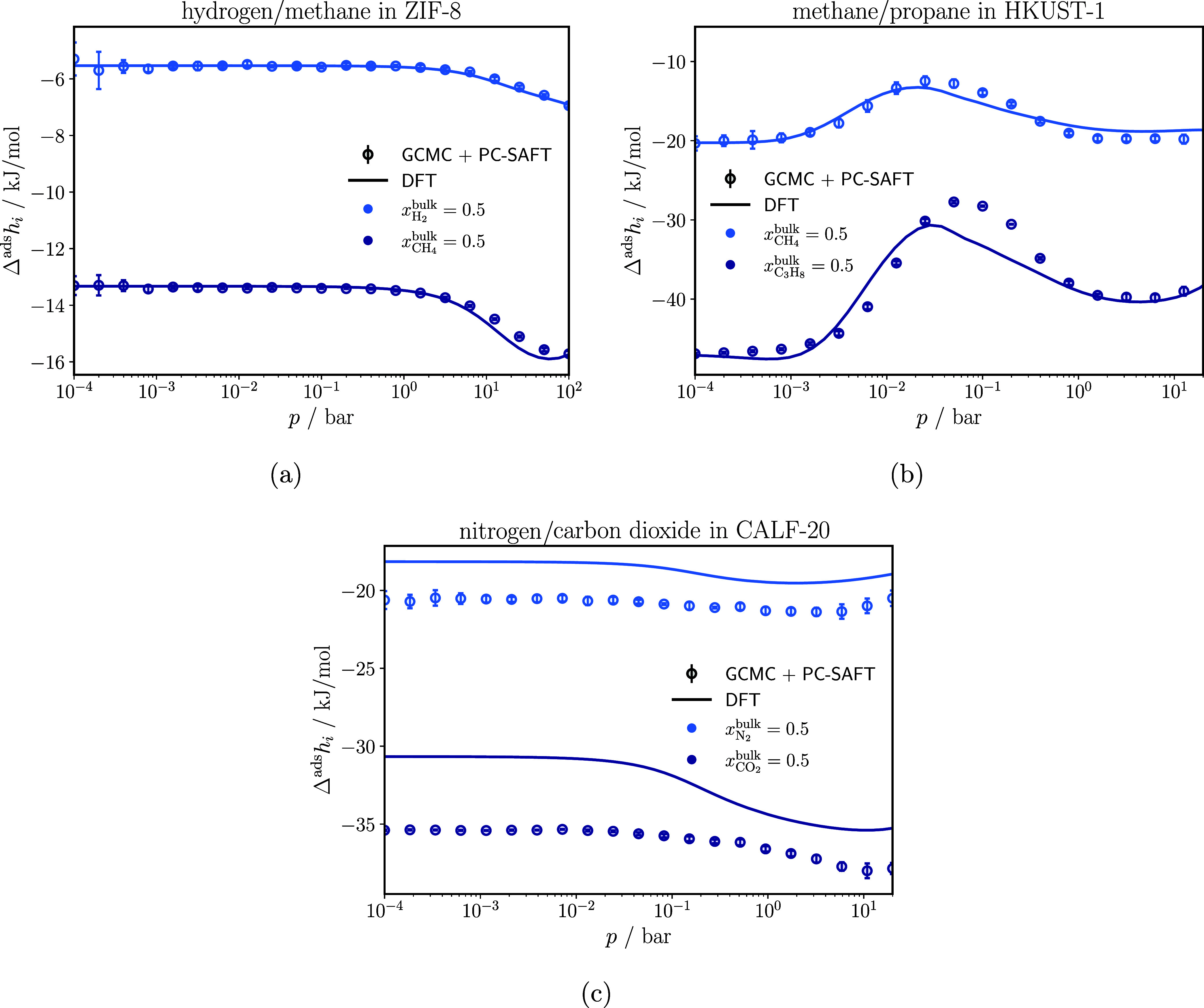
Enthalpy of adsorption for each component of a methane/hydrogen mixture in ZIF-8 (a), of a methane/propane mixture in HKUST-1 (b), and of a carbon dioxide/nitrogen mixture in CALF-20 (c). All enthalpies of adsorption were obtained at 298 K.

Figure 10 shows the comparison of the enthalpy of adsorption of each component in the multicomponent mixtures argon/krypton/xenon and methane/ethane/propane/nitrogen in HKUST-1, calculated with classical DFT and obtained from GCMC + PC-SAFT. For the ternary noble gas mixture, the enthalpies of adsorption calculated with classical DFT and obtained from GCMC + PC-SAFT show good agreement. Classical DFT slightly underestimates the enthalpy of adsorption of all components within the pressure range of 1 bar to 1 × 10^1^ bar, and then slightly overestimates it. For the quaternary mixture, classical DFT reproduces the enthalpy of adsorption for methane and nitrogen well. Compared to GCMC + PC-SAFT, classical DFT slightly underestimates the enthalpy of adsorption in the pressure range from 1 bar to 1 × 10^2^ bar, for ethane and propane.

**Figure 10:**
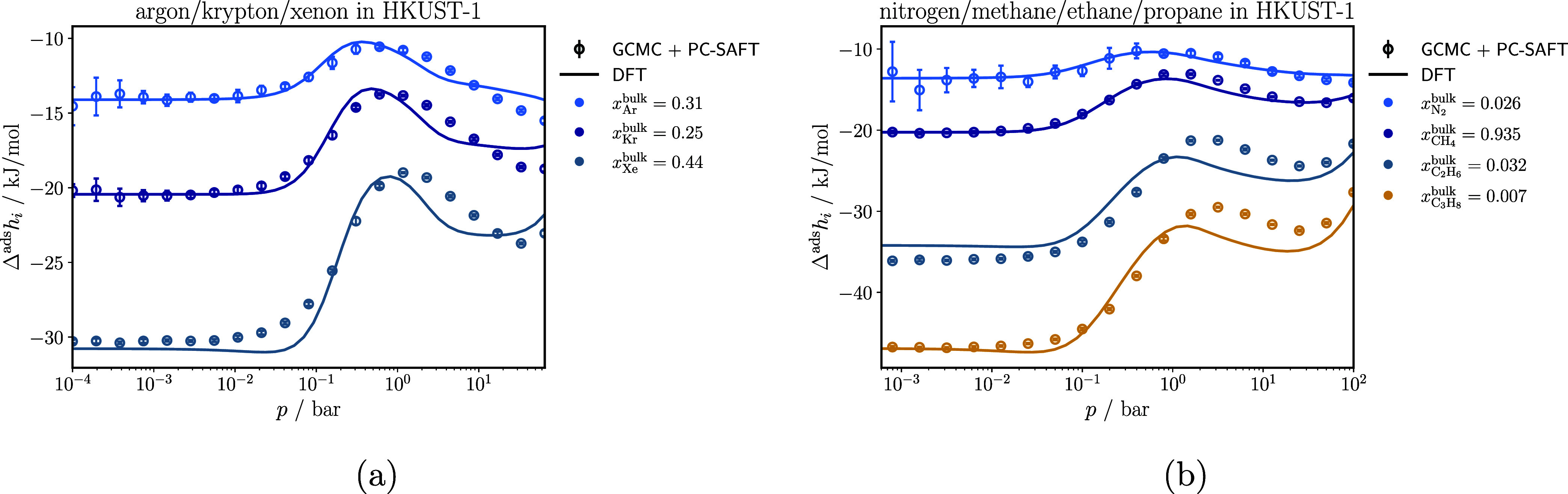
Enthalpy of adsorption for each component of a ternary argon/krypton/xenon mixture (a) and of a quaternary methane/ethane/propane/nitrogen mixture (b), both at 298 K in HKUST-1.

## Conclusion

In this work, we demonstrate that classical DFT, based on the PC-SAFT equation of state, can accurately and efficiently predict adsorption phenomena in mixtures. We compare the predictions from classical DFT to data from own GCMC simulations to ensure a consistent comparability. We perform the analysis across a wide range of systems, including practically relevant binary, ternary, and quaternary gas mixtures at 298 K. In all cases, classical DFT reliably reproduces the absolute loadings and adsorption enthalpies of the mixture components from GCMC calculations.

A strength of classical DFT is its predictive nature. All calculations were performed without relying on input from simulation data or adjustments to the model parameters of the inhomogeneous adsorption system. Instead, the functionals, based on the PC-SAFT equation of state, provide a molecular grounded description of the fluid phase that can be directly applied to adsorption in porous materials. Despite the simplicity of the functionals used, the results matched those of the GCMC simulations closely, demonstrating the robustness of the approach. Classical DFT predicts adsorption isotherms well for spherical and small nonpolar molecules. However, deviations appear for fluids or adsorbents with strong polar or Coulombic interactions, such as CO_2_ especially at low pressure conditions, and deviations are expected for systems involving long-chain molecules (beyond propane).

Using a GPU-accelerated implementation, adsorption isotherms can be obtained in seconds, corresponding to a speedup of several orders of magnitude compared to GCMC simulations. This significant reduction in computational cost enables applications requiring large numbers of adsorption calculations, such as high-throughput screening, optimization of framework structures, and designing adsorbents for specific separation tasks. Classical DFT performs similarly well for binary, ternary, and quaternary mixtures, which opens the possibility of screening more realistic multicomponent systems that are often simplified to binary mixtures. These results establish classical DFT, based on the PC-SAFT equation of state, as a powerful and reliable tool for the prediction of mixture adsorption in porous materials.

## Supplementary Material



## Data Availability

Data for this article, including RASPA input files of the example systems used in this work can be retrieved from the Data Repository of the University of Stuttgart (DaRUS) at https://doi.org/10.18419/DARUS-5542.

## Notes

PC-SAFT and solid atoms Lennard-Jones parameters; pure component adsorption isotherms of all investigated systems (Tables S1, S2, S3 and Figures S1, S2, S3, and S4); multicomponent adsorption isotherms of all investigated systems (Figures S5–S8); computing bulk enthalpies using the isobaric-isothermal ensemble; enthalpy of adsorption of all investigated systems (Figures S9–S13) (PDF)
