# Supplementary material for: Efficient Prediction of Multicomponent Adsorption Isotherms and Enthalpies of Adsorption in MOFs Using Classical Density Functional Theory
Source: J Phys Chem B. 2026 Feb 5;130(7):2299–313. doi: 10.1021/acs.jpcb.5c08035 (PMC12926958; doi:10.1021/acs.jpcb.5c08035)
Supplement: Supplementary file 1 [file jp5c08035_si_001.pdf]

Supporting Information for: Efficient Prediction of Multicomponent Adsorption Isotherms and Enthalpies of Adsorption in MOFs Using Classical Density Functional Theory

Nadine Thiele,[1] Tiong Wei Teh,[1] Benjamin Bursik,[1] Marcel Granderath,[2] Gernot Bauer,[1] Vincent Dufour-Décieux,[2] Philipp Rehner,[2] Rolf Stierle,[1] André Bardow,[2] Niels Hansen,[1] and Joachim Gross[1, a)]

[1)] *Institute of Thermodynamics and Thermal Process Engineering, University of Stuttgart, Pfaffenwaldring 9, 70569 Stuttgart, Germany*

[2)] *Energy & Process Systems Engineering, Department of Mechanical and Process Engineering, ETH Zurich, Tannenstrasse 3, 8092 Zurich, Switzerland*

1. PURE COMPONENT ADSORPTION ISOTHERMS OF ALL INVESTIGATED SYSTEMS

Table S1. Parameters for the PC-SAFT equation of state.

Fluid	m_i	σ_{ii}/Å	$\frac{\varepsilon_{ii}}{k_B}$/K	Ref.
H_2	1	2.960	34.20	Buch [1]
Ar	1	3.378	117.81	Esper *et al.* [2]
Kr	1	3.608	164.02	Esper *et al.* [2]
Xe	1	3.927	227.70	Esper *et al.* [2]
CH_4	1	3.701	150.07	Esper *et al.* [2]
C_2H_6	1.607	3.517	191.45	Esper *et al.* [2]
C_3H_8	1.986	3.624	209.09	Esper *et al.* [2]
N_2	1.238	3.300	89.41	Esper *et al.* [2]
CO_2	2.531	2.579	153.32	Esper *et al.* [2]

Table S2. Binary interaction parameters for the PC-SAFT equation of state, adjusted to vapor-liquid equilibrium data by Rehner, Bardow, and Gross [3]

Fluid i	Fluid j	k_{ij}
CH_4	C_2H_6	−0.004 894 18
CH_4	C_3H_8	−0.005 506 77
C_2H_6	C_3H_8	0.012 499 41

Table S3. Lennard–Jones potential parameters for the solid atoms in the investigated frameworks derived from the UFF[4] and DREIDING[5] force fields.

solid atom	σ_{ss}/Å	$\frac{\varepsilon_{ss}}{k_B}$/K	force field
C	3.472 99	47.8562	DREIDING[5]
H	2.846 42	7.648 938	DREIDING[5]
N	3.262 56	38.9492	DREIDING[5]
O	3.033 15	48.1581	DREIDING[5]
Zn	2.461 55	62.399 23	UFF[4]
Cu	3.113 69	2.5161	UFF[4]
V	2.800 99	8.051 51	UFF[4]

[a)] Electronic mail: joachim.gross@itt.uni-stuttgart.de

Figure S2 shows the unit cells of all MOFs considered in this study. Figures S3 and S4 show the adsorption isotherms of the pure components of all the mixtures investigated and listed in Table 2. These adsorption isotherms were obtained using GCMC simulations and classical DFT calculations. The PC-SAFT parameters used in this work are listed in Table S1. The binary interaction parameters for the PC-SAFT model employed in this work are provided in Table S2. In this work, parameters from the Universal Force Field (UFF)[4] and DREIDING[5] are employed, given in Table S3.

Slight discrepancies appear when the adsorption isotherms of xenon are compared (see Figure S3d and e). Therefore, the vapor-liquid equilibrium (VLE) is examined. Figure S1 shows the comparison of the VLE data for xenon from NIST[6] with PC-SAFT and with the force field provided by Vrabec, Stoll, and Hasse [7].

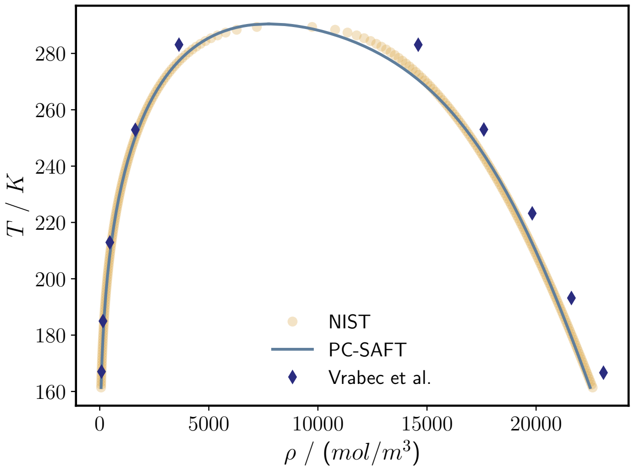

Figure S1. Vapor-liquid equilibrium data of xenon obtained from NIST[6], PC-SAFT and Vrabec, Stoll, and Hasse [7].

2. MULTICOMPONENT ADSORPTION ISOTHERMS OF ALL INVESTIGATED SYSTEMS

Figures S5 and S6 show the adsorption isotherms of the systems listed in Table 2 at 298 K. These adsorption isotherms were obtained from GCMC simulations, classical DFT calculations, and from IAST with

pure component GCMC simulations as input (IAST + GCMC). To highlight the low-pressure regime, all adsorption isotherms are shown in a double-logarithmic diagram (see Figures S7 and S8).

3. COMPUTING BULK ENTHALPIES USING THE ISOBARIC-ISOTHERMAL ENSEMBLE

To calculate the partial molar enthalpy of adsorption, $\Delta h_i^{\mathrm{ads}}$, from molecular simulations, it is necessary to account for the bulk enthalpy, following the approach in previous work[8]. We conduct NPT simulations for pure components. The NPT bulk simulations consist of 5×10^4 equilibration cycles, followed by 5×10^5 production cycles for $N = 256$ molecules. The initial box lengths are estimated from the bulk density, ρ^{bulk}, obtained using the PC-SAFT equation of state. Translation, rotation, regrow, and volume-change moves were performed in the ratio $(1 : 1 : 1 : 0.1)$.

4. ENTHALPY OF ADSORPTION OF ALL INVESTIGATED SYSTEMS

Figures S9 and S10 show a comparison of the enthalpies of adsorption for all pure components, obtained from PC-SAFT, GCMC + NPT, and GCMC + PC-SAFT. Figures S11–S13 show the enthalpies of adsorption for the binary, ternary, and quaternary mixtures.

5. ADDITIONAL MATERIAL

Data for this article, including RASPA input files of the example systems used in this work can be retrieved from the Data Repository of the University of Stuttgart (DaRUS) at https://doi.org/10.18419/DARUS-5542

6. REFERENCES

[1] V. Buch, “Path integral simulations of mixed para-D2 and ortho-D2 clusters: The orientational effects,” The Journal of Chemical Physics **100**, 7610–7629 (1994).

[2] T. Esper, G. Bauer, P. Rehner, and J. Gross, “PCP-SAFT Parameters of Pure Substances Using Large Experimental Databases,” Industrial & Engineering Chemistry Research **62**, 15300–15310 (2023).

[3] P. Rehner, A. Bardow, and J. Gross, “Modeling Mixtures with PCP-SAFT: Insights from Large-Scale Parametrization and Group-Contribution Method for Binary Interaction Parameters,” International Journal of Thermophysics **44** (2023), 10.1007/s10765-023-03290-3.

[4] A. K. Rappe, C. J. Casewit, K. S. Colwell, W. A. Goddard, and W. M. Skiff, “UFF, a full periodic table force field for molecular mechanics and molecular dynamics simulations,” Journal of the American Chemical Society **114**, 10024–10035 (1992).

[5] S. L. Mayo, B. D. Olafson, and W. A. Goddard, “DREIDING: a generic force field for molecular simulations,” The Journal of Physical Chemistry **94**, 8897–8909 (1990).

[6] P. Linstrom, en“NIST Chemistry WebBook, NIST Standard Reference Database 69,” (1997).

[7] J. Vrabec, J. Stoll, and H. Hasse, “A set of molecular models for symmetric quadrupolar fluids,” The Journal of Physical Chemistry B **105**, 12126–12133 (2001).

[8] T. W. Teh, P. Franz, R. Stierle, N. Hansen, and J. Gross, “Classical density functional theory for alkane adsorption in cationic Faujasites: comparison with grand canonical Monte Carlo simulations,” Molecular Physics , e2471510 (2025).

[9] D. Dubbeldam, S. Calero, and T. J. Vlugt, “iRASPA: GPU-accelerated visualization software for materials scientists,” Molecular Simulation **44**, 653–676 (2018).

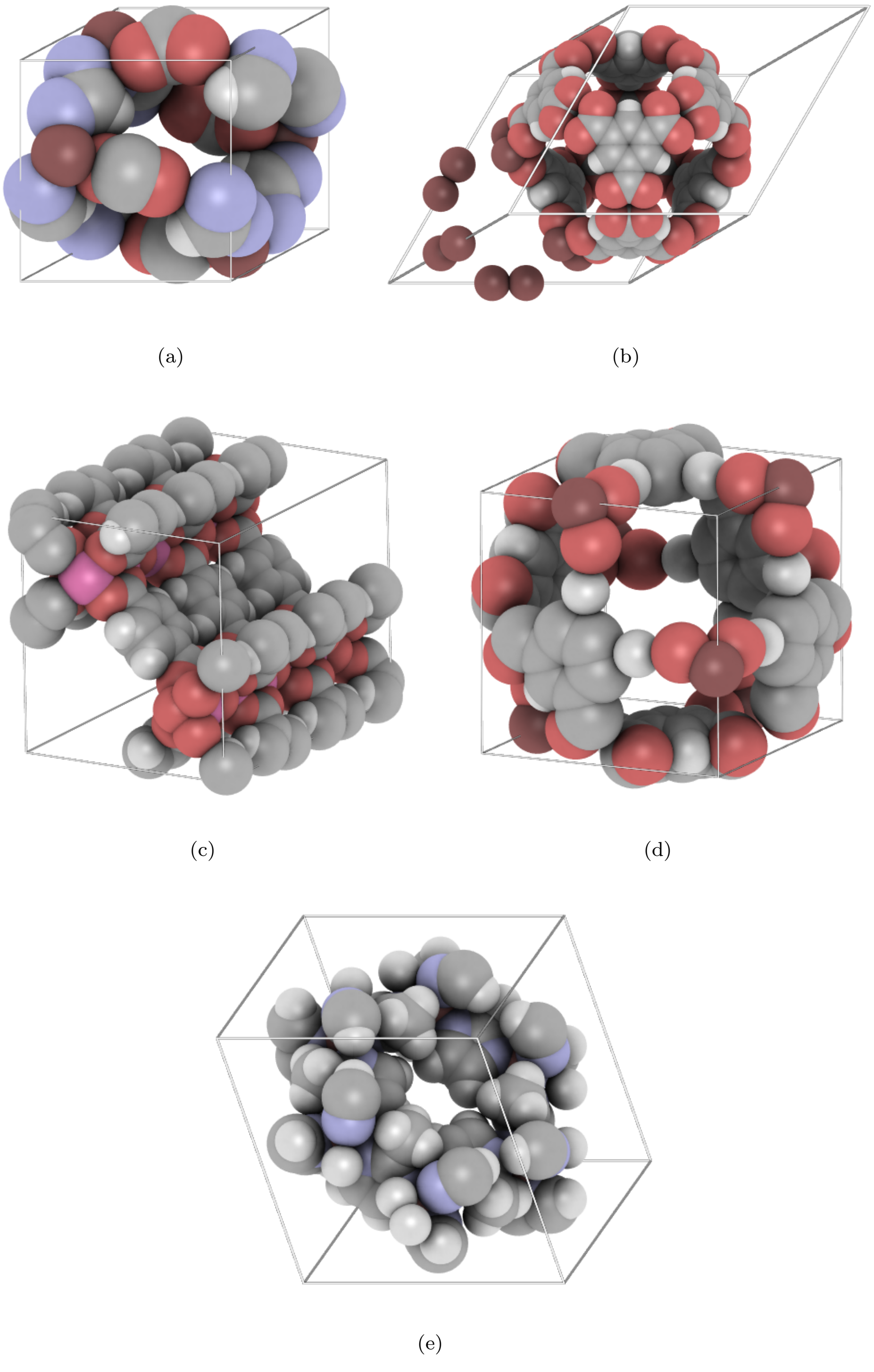

Figure S2. Unit cell of CALF-20 (a), HKUST-1 (b), MIL-47 (c), MOF-505 (d), and ZIF-8 (e), visualized with iRASPA.[9]

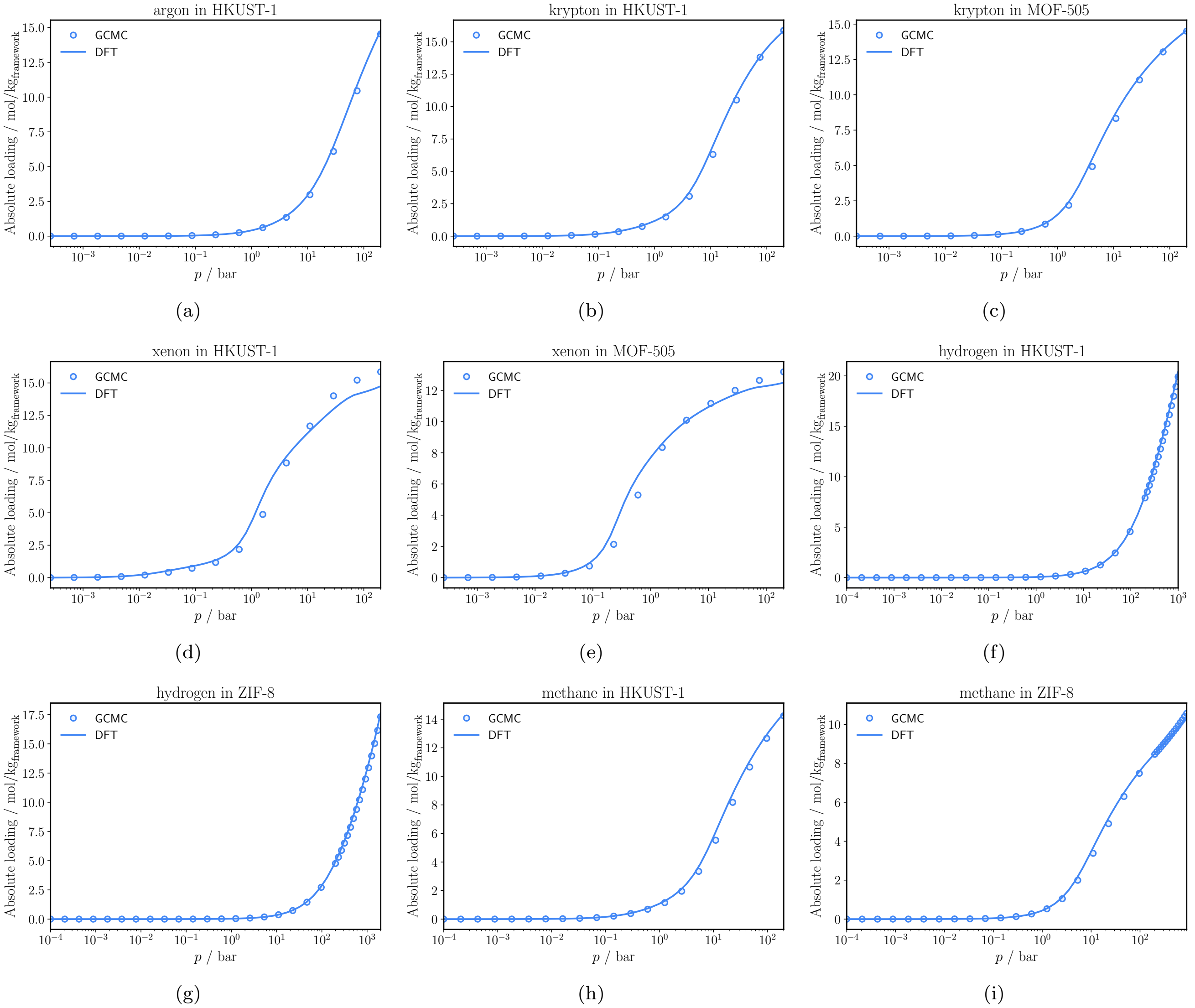

Figure S3. Pure component adsorption isotherms of argon (a), krypton (b, c), xenon (d, e), hydrogen (f, g), and methane (h, i) in different solid materials at 298 K.

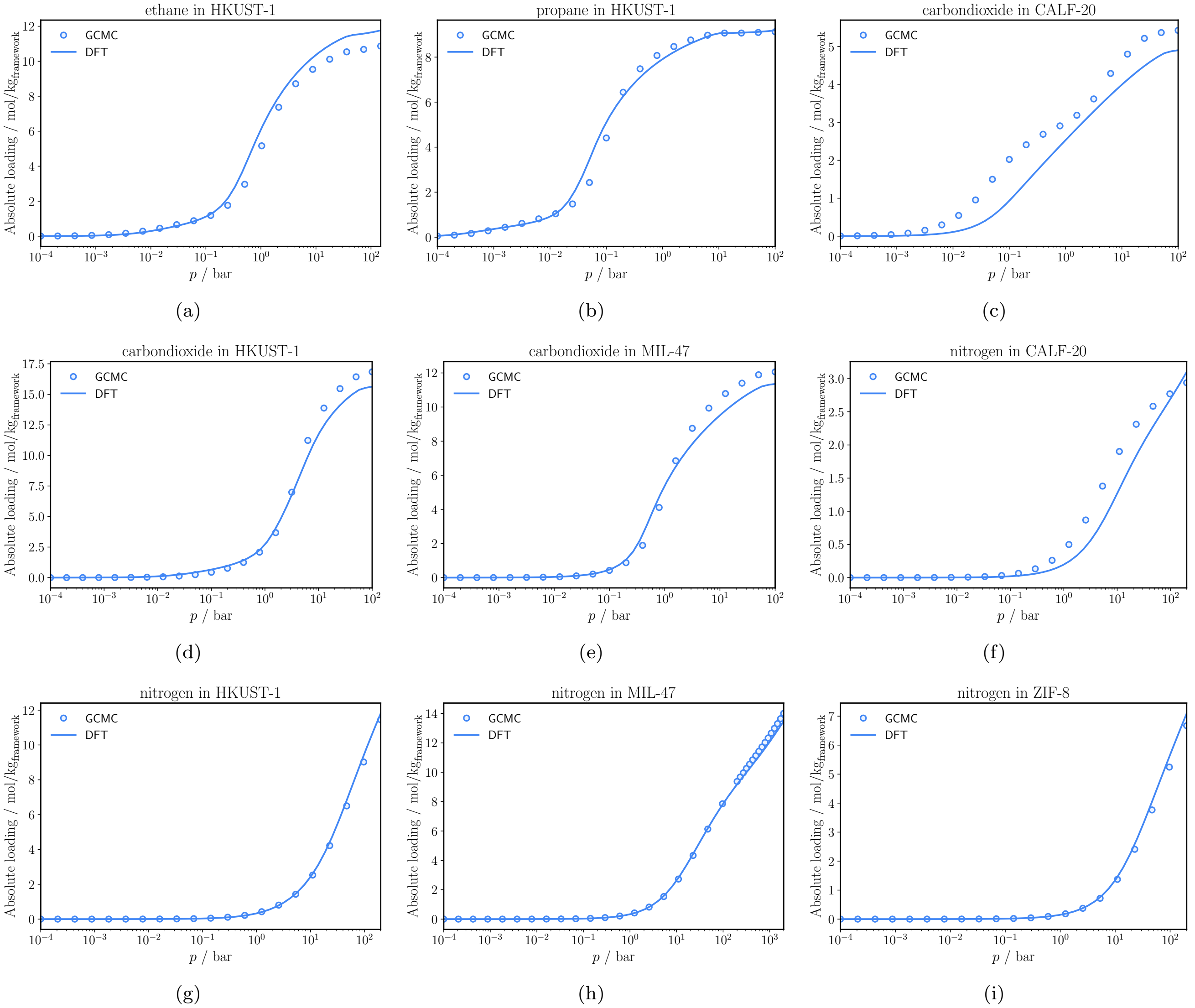

Figure S4. Pure component adsorption isotherms of ethane (a), propane (b), carbon dioxide (c - e), and nitrogen (f - i) in different solid materials at 298 K.

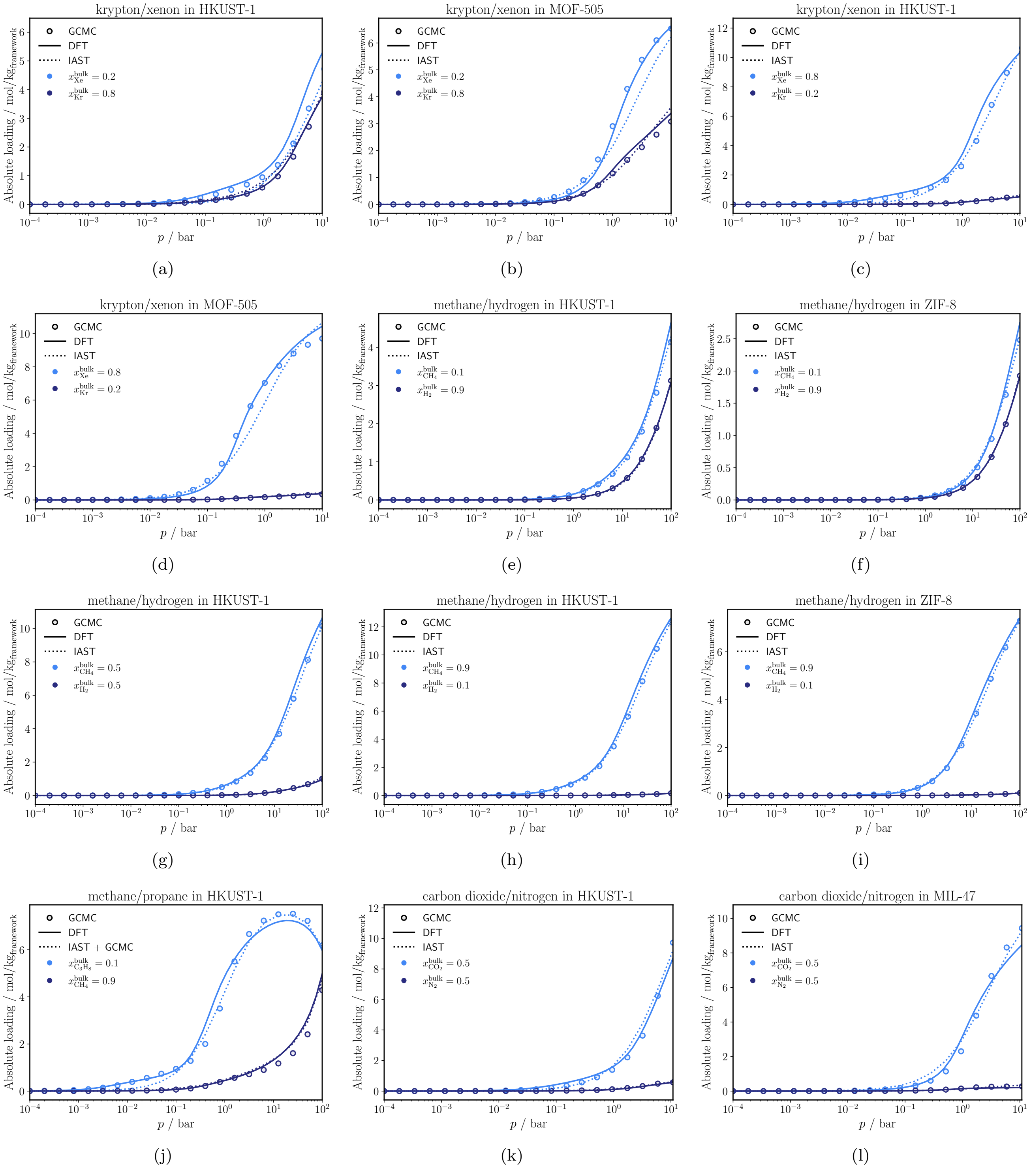

Figure S5. Adsorption isotherms of krypton/xenon (a - d), methane/hydrogen (e - i), methane/propane (j), and carbon dioxide/nitrogen (k,l) in different solid materials at 298 K. The IAST results are based on Langmuir-fits on the pure component GCMC isotherms. The statistical uncertainties in the GCMC results are smaller than the symbol size.

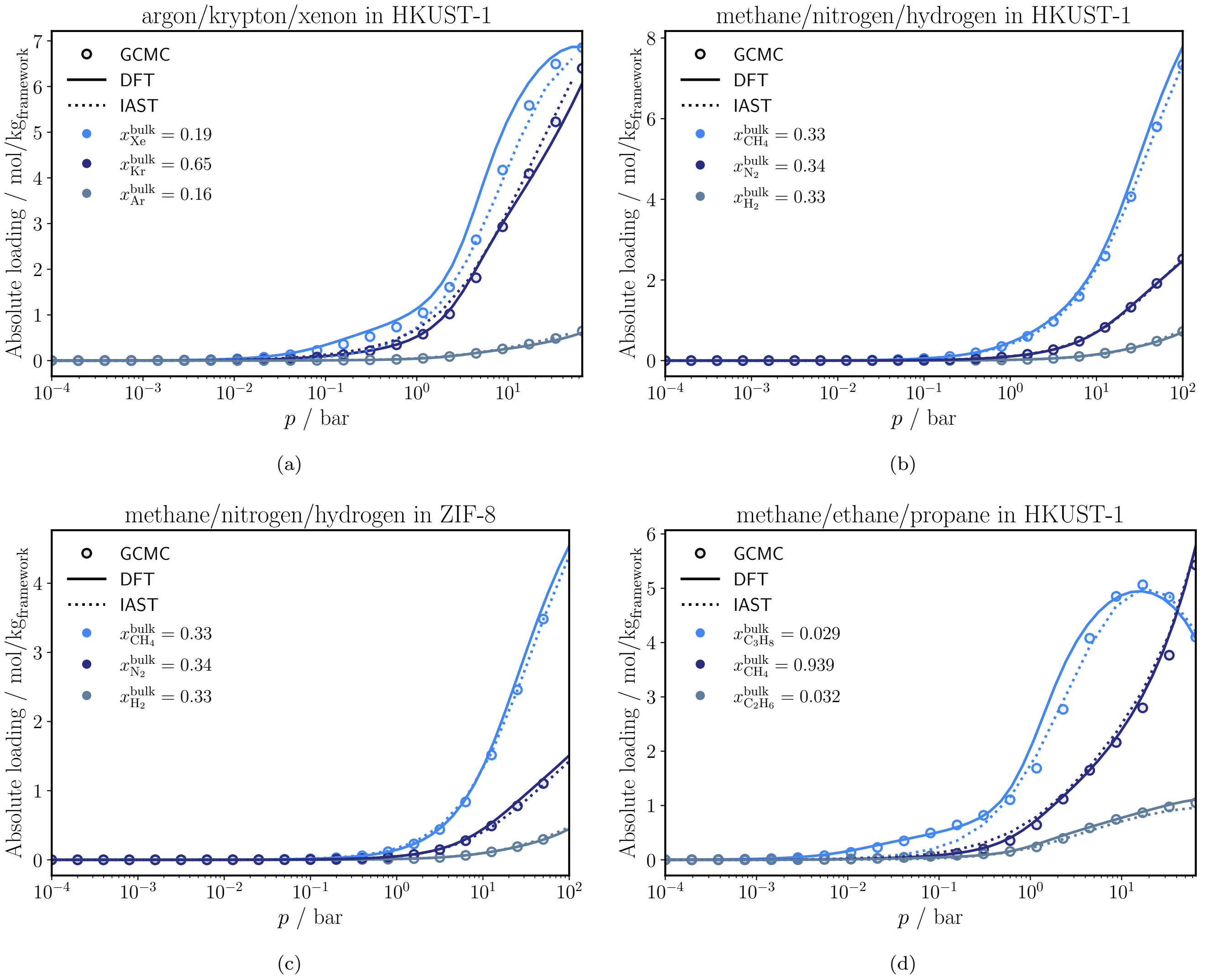

Figure S6. Adsorption isotherms of argon/krypton/xenon (a), methane/hydrogen/nitrogen (b, c), and methane/ethane/propane (d) in different solid materials at 298 K. The IAST results are based on Langmuir-fits on the pure component GCMC isotherms. The statistical uncertainties in the GCMC results are smaller than the symbol size.

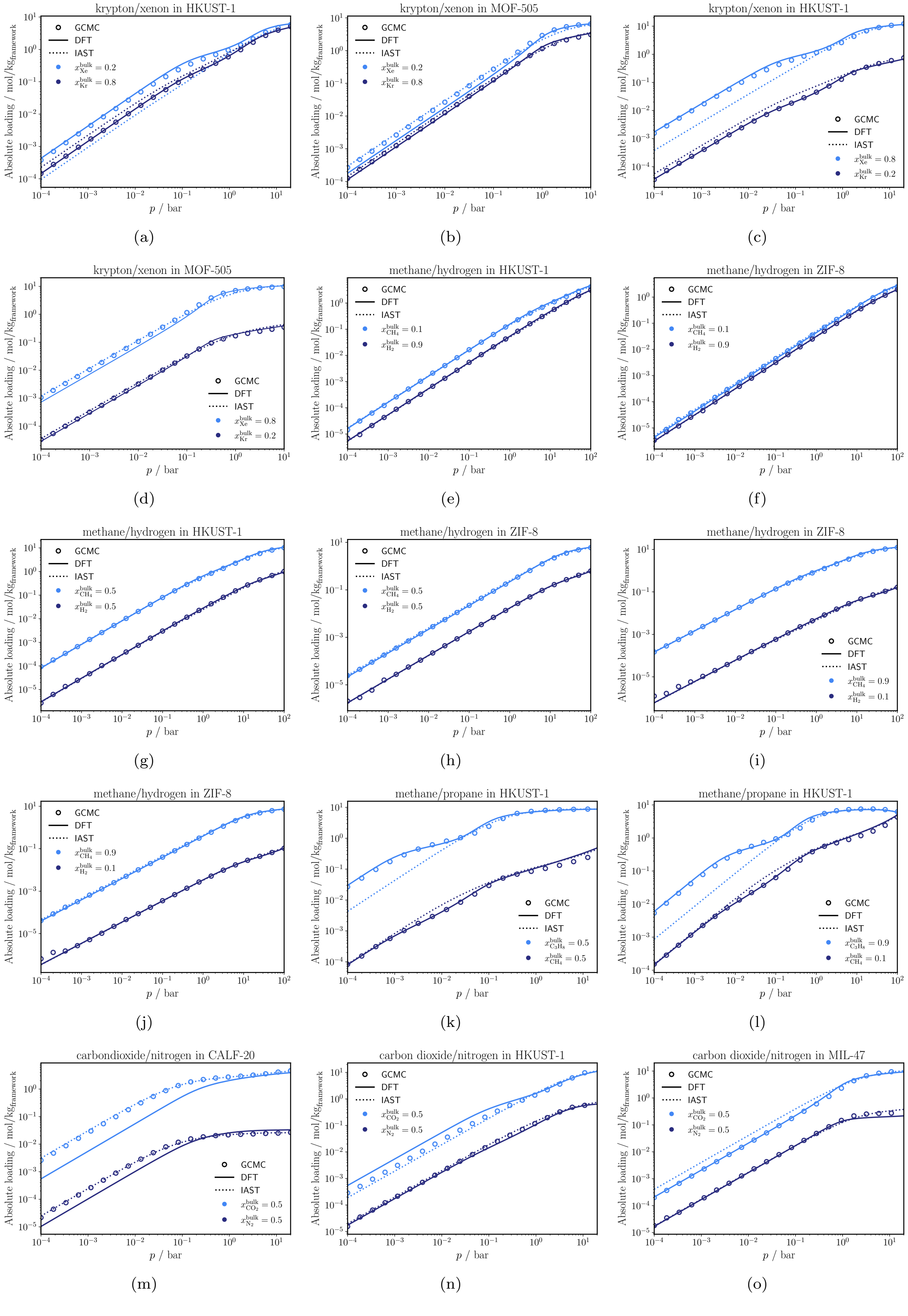

Figure S7. Adsorption isotherms of krypton/xenon (a - d), methane/hydrogen (e - j), methane/propane (k,l), and carbon dioxide/nitrogen (m - o) in different solid materials at 298 K. The IAST results are based on Langmuir-fits on the pure component GCMC isotherms. The statistical uncertainties in the GCMC results are smaller than the symbol size.

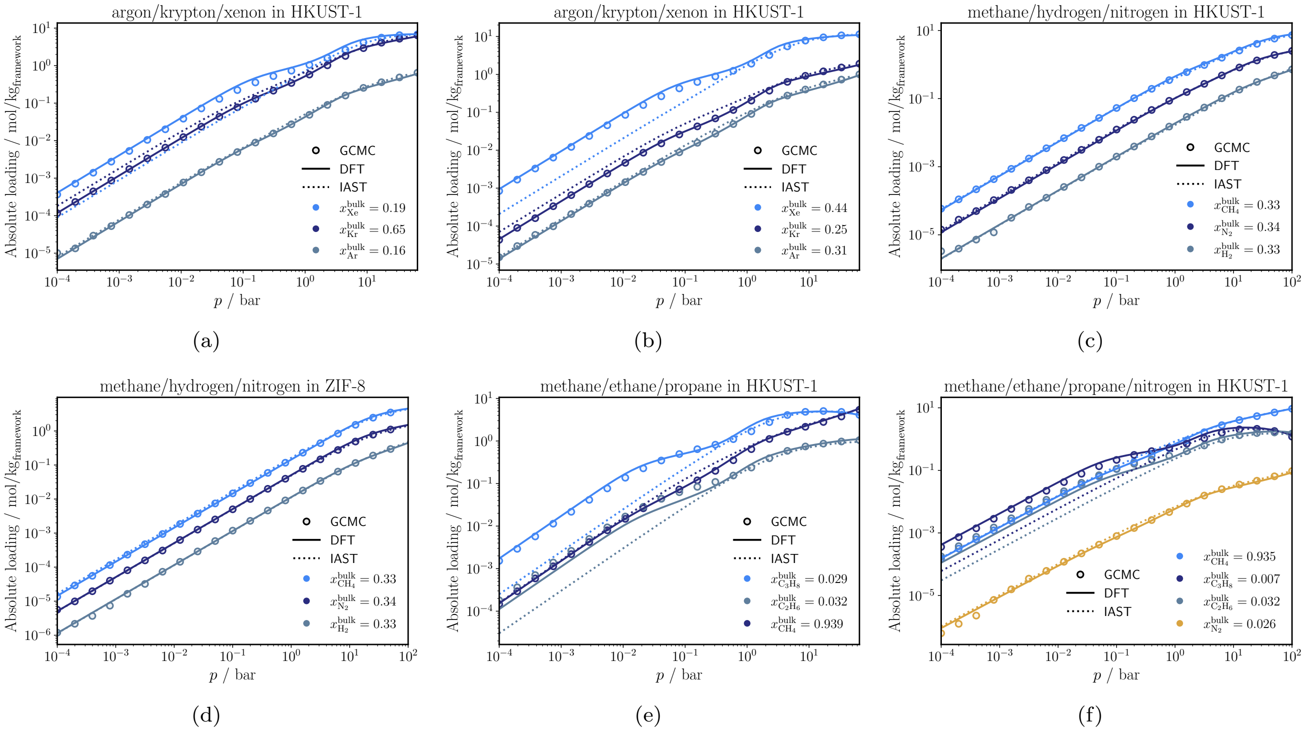

Figure S8. Adsorption isotherms of argon/krypton/xenon (a,b), methane/hydrogen/nitrogen (c, d), methane/ethane/propane (e), and methane/ethane/propane/nitrogen (f) in different solid materials at 298 K. The IAST results are based on Langmuir-fits on the pure component GCMC isotherms. The statistical uncertainties in the GCMC results are smaller than the symbol size.

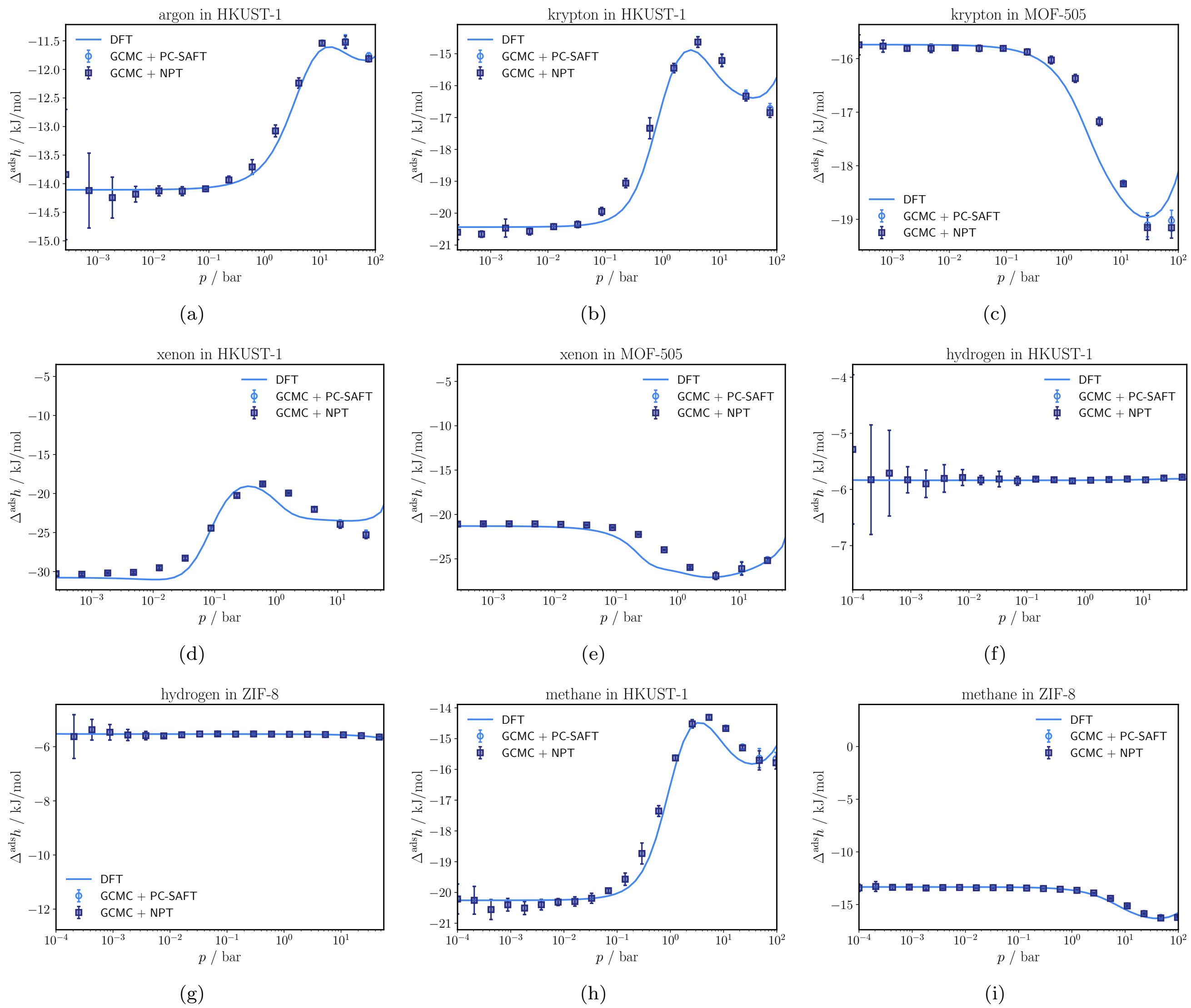

Figure S9. Enthalpies of adsorption of argon (a), krypton (b, c), xenon (d, e), hydrogen (f, g), and methane (h, i) in different solid materials at 298 K.

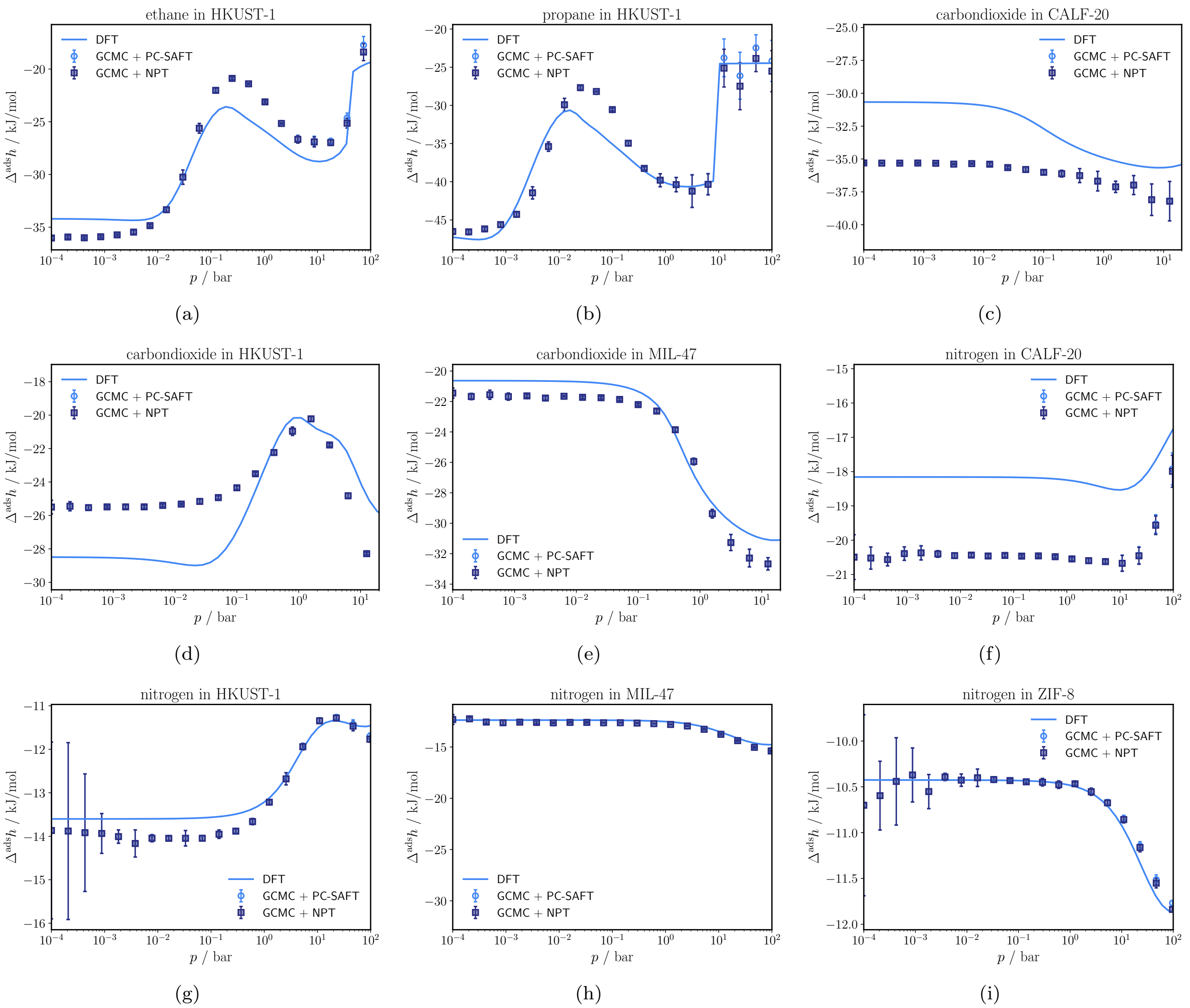

Figure S10. Enthalpies of adsorption of ethane (a), propane (b), carbon dioxide (c - e), and nitrogen (f - i) in different solid materials at 298 K.

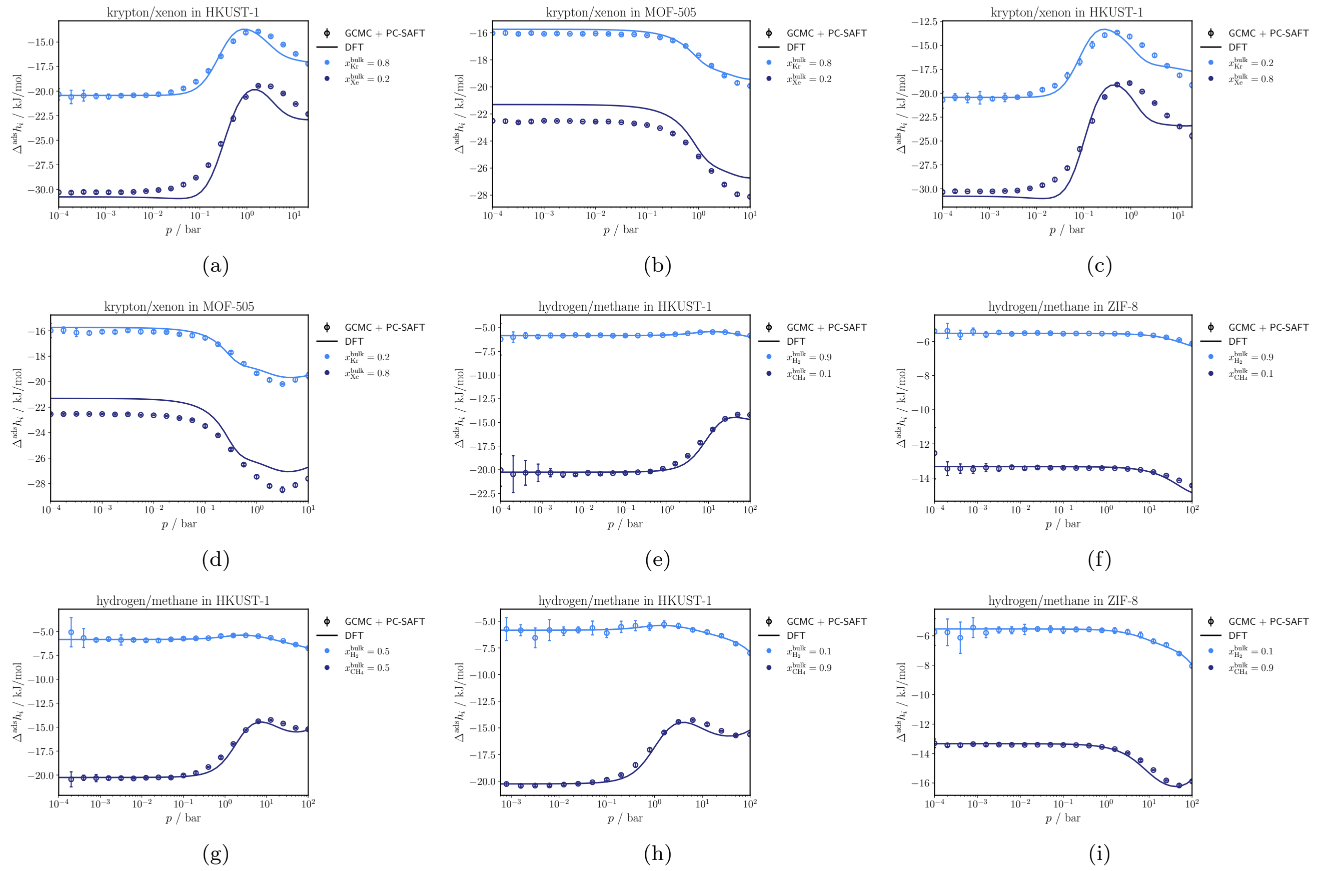

Figure S11. Enthalpies of adsorption of krypton/xenon (a - d), and methane/hydrogen (e - i) in different solid materials at 298 K.

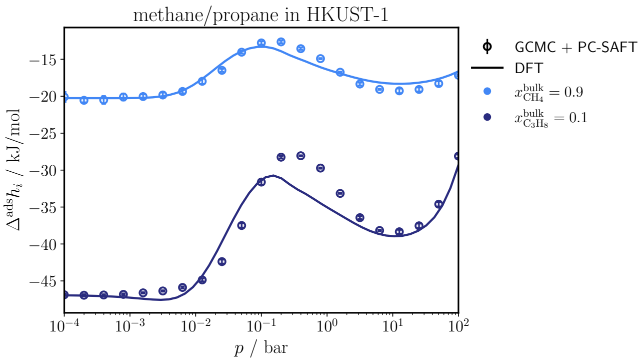

(a)

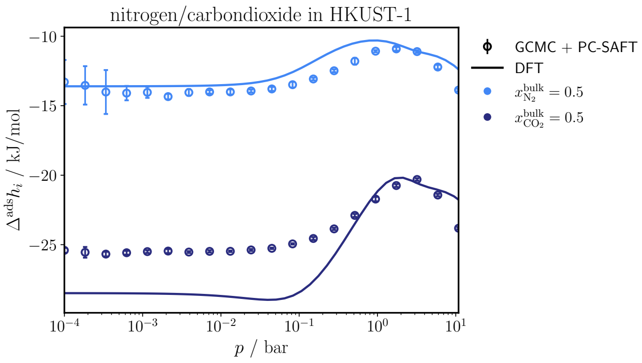

(b)

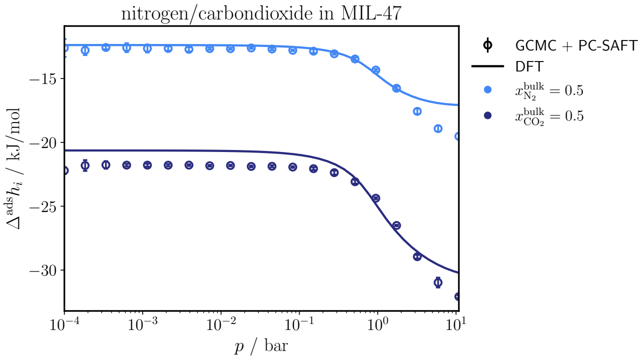

(c)

Figure S12. Enthalpies of adsorption of methane/propane (a), and carbon dioxide/nitrogen (b,c) in different solid materials at 298 K.

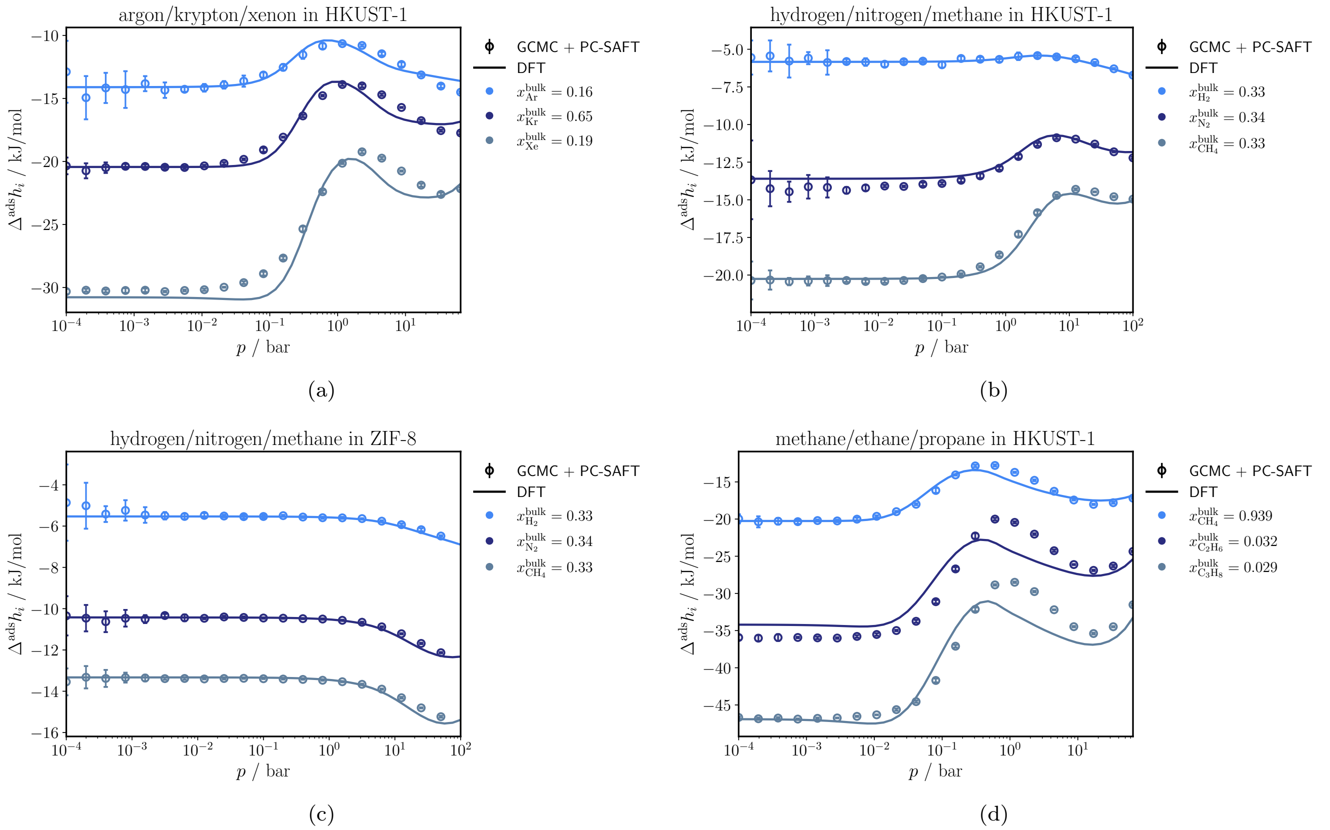

Figure S13. Enthalpies of adsorption of argon/krypton/xenon (a), methane/hydrogen/nitrogen (b, c), and methane/ethane/propane (d) in different solid materials at 298 K.
